# Genomic plasticity and mobilome architecture of Vibrio europaeus reveal key mechanisms of evolutionary adaptation

**DOI:** 10.1099/mgen.0.001600

**Published:** 2026-01-21

**Authors:** Sergio Rodriguez, Diego Rey-Varela, Clara Martinez, Paulino Martinez, Marie-Agnès Travers, Juan L. Barja, Javier Dubert

**Affiliations:** 1 Aquatic One Health Research Center (iARCUS), Department of Microbiology and Parasitology, CIBUS bldg – Faculty of Biology, University of Santiago de Compostela, Santiago de Compostela, Spain; 2 Department of Genetics, Faculty of Veterinary, University of Santiago de Compostela, Lugo, Spain; 3 Ifremer, ASIM, La Tremblade, France; 4 IHPE, Université de Montpellier, CNRS, Ifremer, Université de Perpignan Via Domitia, Montpellier, France

**Keywords:** accessory genome, anti-phage defence system, horizontal gene transfer (HGT), mobilome, regions of genomic plasticity (RGPs), *Vibrio europaeus*

## Abstract

*Vibrio europaeus* has emerged as a significant pathogen in shellfish aquaculture, causing mass mortality outbreaks in key bivalve species and leading to severe economic losses for the industry. Studies on the structure and characteristics of the accessory genome in aquaculture pathogens remain scarce, despite its crucial role in evolutionary and ecological adaptation. The accessory genome provides indeed genetic variability that enables rapid responses to environmental challenges, host adaptation and selective pressures such as antibiotics or phage predation. Here, we present the first comprehensive comparative genomic analysis of the *V. europaeus* pangenome to investigate the structural organization and functional content of its accessory genome. The soft mobilome of *V. europaeus* comprises 73% of accessory genes and 44% of the total pangenome, including non-chromosomic (plasmids) and chromosomic genetic elements such as prophages, integrative and conjugative/mobilizable elements, phage satellites and other mobile genetic elements (MGEs) designated as unclassified chromosomic regions of genomic plasticity (unclassified chromosomic RGPs). Among accessory elements, unclassified chromosomic RGPs were the primary drivers of evolutionary dynamics in *V. europaeus*, acting as the main genetic reservoir of anti-phage defence systems and antimicrobial resistance genes. Notably, the identification of abundant insertion hotspots in chromosomic genetic elements facilitates the rapid acquisition of anti-phage defence systems, thereby enabling rapid turnover of these systems and enhancing host fitness. In addition, novel pVE1-like plasmids (>300 kb) – only found in this species and its closest relative *Vibrio tubiashii* – emerged as the largest and most ubiquitous MGEs in *V. europaeus*. These plasmids encode the highest number of virulence genes and secondary metabolite biosynthetic genes, as well as a remarkable diversity of anti-phage defence systems among closely related strains. Although the genome dataset analysed here is limited to strains isolated from moribund/dead animals in aquaculture environments, this study provides new insights into the role of accessory genetic elements in the evolution, adaptation and diversification of the shellfish pathogen *V. europaeus*. The findings reveal the complexity and plasticity of its pangenome and highlight the importance of RGPs and plasmids in bacterial fitness.

**Impact Statement**

This work presents the first study about the structural organization of the *Vibrio europaeus* accessory genome. Research on accessory genome structure in non-clinical *Vibrio* spp. remains scarce, despite its pivotal role in evolutionary and ecological adaptation and its capacity to reshape bacterial communities through horizontal gene transfer. This is particularly relevant in * V. europaeus*, where the pangenome is mostly represented by the accessory genome (61%). Our analysis approached the accessory genome from the perspective of mobile genetic elements (MGEs), including plasmids, prophages, integrative and conjugative/mobilizable elements, phage satellites and especially unclassified chromosomic regions of genomic plasticity. From a functional standpoint, the study revealed that while virulence factors and secondary metabolite clusters contribute to turnover, anti-phage defence systems are the most dynamic feature, driving strain diversification and shaping population structure. Comparative analyses of anti-phage defence systems provided insights into the evolutionary trajectories of *V. europaeus* strains across diverse hosts, locations and outbreaks, highlighting their utility as tools for population dynamics studies. In addition, we identified and characterized a novel large plasmid family, the pVE1 plasmids (>300 kb), which are ubiquitous in this species and encode a remarkable repertoire of virulence, secondary metabolite and anti-phage defence systems, underlining their major role of a plasmid in *V. europaeus* biology. All this knowledge uncovered the complexity and plasticity of the *V. europaeus* accessory genome, providing new insights into its role in adaptation and evolution, and will be of particular interest to researchers studying MGEs in *Vibrio* spp. and other marine bacterial communities.

## Data Summary

All genome assemblies have been uploaded to the National Center for Biotechnology Information. The GenBank accession numbers for each of the 39 strains used in this study, and detailed information can be found in Table 1. All bioinformatics tools used for comparative genomics have been listed in the Methods section, including references, associated databases and analysis parameters.

**Table 1.** *V. europaeus* (*n*=39) genomes used in this study Only the strain 071316F was not associated with mortality outbreaks.

Strain	Assembly*	Accession no.	Location	Year	Source	Phylogenetic cluster†
EX1	comp (4)	CP180205-CP180207	Hatchery A (Spain)	July 1985	*Ostrea edulis* larvae	VII
PP-654	cont (38)	JAPFJT000000000.1	Hatchery B (Spain)	March 2001	*O. edulis* larvae	Ia
PP-660	cont (34)	JAPFJS000000000.1	Hatchery B (Spain)	March 2001	*O. edulis* larvae	Ia
PP-635	cont (38)	JAPFJR000000000.1	Hatchery B (Spain)	March 2001	*O. edulis* seawater tank	Ia
CECT8136T	comp (4)	LUAX00000000.1	Hatchery B (Spain)	March 2001	*O. edulis* seawater tank	Ia
CECT8427	comp (4)	JAPFJQ000000000.1	Hatchery C (France)	January 2004	*Haliotis tuberculata* spat	III
CECT8426	comp (3)	GCA_015654285.1	Hatchery D (France)	June 2007	*Magallana gigas* spat	IIb
07/038 2T2	cont (44)	JAPFJP000000000.1	Hatchery D (France)	August 2007	*M. gigas* spat	IV
07/108 T1	cont (43)	JAPFJO000000000.1	Hatchery D (France)	August 2007	*M. gigas* spat	VIII
07/110 T1	cont (43)	JAPFJN000000000.1	Hatchery D (France)	August 2007	*M. gigas* spat	IIa
07/112 T1	cont (36)	JAPFJM000000000.1	Hatchery D (France)	August 2007	*M. gigas* spat	IIa
07/115 T2	cont (47)	JAPFJL000000000.1	Hatchery D (France)	August 2007	*M. gigas* spat	VI
07/116 T1	cont (37)	JAPFJK000000000.1	Hatchery D (France)	August 2007	*M. gigas* spat	IIa
07/117 T1	cont (37)	JAPFJJ000000000.1	Hatchery D (France)	August 2007	*M. gigas* spat	III
07/120 T1	cont (41)	JAPFJI000000000.1	Hatchery D (France)	August 2007	*M. gigas* spat	IIb
07/121 1T1	cont (39)	JAPFJH000000000.1	Hatchery D (France)	August 2007	*M. gigas* spat	IIa
PP2-843	comp (4)	GCA_028447005.1	Hatchery E (Spain)	November 2008	*Ruditapes philippinarum* spat	Ib
PP2-978	cont (32)	JAPFJF000000000.1	Hatchery E (Spain)	November 2008	*R. philippinarum* spat	Ib
2909	cont (34)	JAPFJE000000000.1	Hatchery B (Spain)	May 2011	*Ruditapes decussatus* seawater tank	Ib
2895	cont (33)	JAPFJD000000000.1	Hatchery B (Spain)	May 2011	*R. decussatus* seawater tank	Ia
2930	cont (36)	JAPFJC000000000.1	Hatchery B (Spain)	May 2011	*R. decussatus* larvae	Ia
2951	cont (37)	JAPFJB000000000.1	Hatchery B (Spain)	May 2011	*R. decussatus* larvae	Ia
2945	cont (33)	JAPFJA000000000.1	Hatchery B (Spain)	May 2011	*R. decussatus* seawater tank	Ia
2967	cont (33)	JAPFIZ000000000.1	Hatchery B (Spain)	May 2011	*R. decussatus* larvae	Ib
2968	cont (34)	JAPFIY000000000.1	Hatchery B (Spain)	May 2011	*R. decussatus* seawater tank	Ia
2969	cont (37)	JAPFIX000000000.1	Hatchery B (Spain)	May 2011	*R. decussatus* larvae	Ia
2971	cont (35)	JAPFIW000000000.1	Hatchery B (Spain)	May 2011	*R. decussatus* seawater tank	Ia
2974	cont (39)	JAPFIV000000000.1	Hatchery B (Spain)	May 2011	*R. decussatus* larvae	Ib
2975	cont (35)	JAPFIU000000000.1	Hatchery B (Spain)	May 2011	*R. decussatus* seawater tank	Ia
3454	cont (45)	JAPFIT000000000.1	Hatchery B (Spain)	May 2012	*Donax trunculus* larvae	Ia
3492	cont (45)	JAPFIS000000000.1	Hatchery B (Spain)	June 2012	*D. trunculus* seawater tank	Ia
3610	cont (40)	JAPFIR000000000.1	Hatchery B (Spain)	July 2012	*R. decussatus* broodstock	Ia
3614	cont (40)	JAPFIQ000000000.1	Hatchery B (Spain)	July 2012	*R. decussatus* eggs	Ia
NPI1	comp (3)	GCA_013154935.1	Hatchery F (Chile)	February 2015	*A. purpuratus* larvae	IIc
071316F	cont (85)	VTYH00000000.1	Netarts Bay (USA)	July 2016	Seawater	V
L2	cont (40)	JAPFIP000000000.1	Hatchery B (Spain)	March 2018	*Polititapes rhomboides* larvae	Ib
L3	cont (40)	JAPFIO000000000.1	Hatchery B (Spain)	March 2018	*Ensis magnus* larvae	Ia
L4	cont (36)	JAPFIN000000000.1	Hatchery B (Spain)	March 2018	*E. magnus* larvae	Ia
L20	cont (37)	JAPFIM000000000.1	Hatchery B (Spain)	May 2018	*R. philippinarum* larvae	Ia

*Assembly level: cont (contigs), comp (fully resolved) and number of contigs are depicted in brackets.

†Phylogenetic clusters based on the core-genome phylogeny previously established by Rodriguez *et al.* [8].

## Introduction

Bivalve aquaculture represents the second most important sector within global aquaculture [1]. However, the expansion of bivalve aquaculture is constrained by the negative impact of bacterial diseases, such as vibriosis caused by some pathogenic *Vibrio* species. Vibriosis is a major disease affecting both farmed and wild bivalves, leading to recurrent mass mortalities that threaten the sustainability and economic viability of this sector [2]. Among these pathogens, *Vibrio europaeus* has emerged over the last decade as one of the most important bivalve pathogens [2]. This bacterial species exhibits a broad host range, including the main bivalve aquaculture species – such as the Pacific oyster (*Magallana gigas*), Manila clam (*Ruditapes philippinarum*) and Chilean scallop (*Argopecten purpuratus*), among others – and affects multiple stages of the bivalve life cycle, including larvae, post-larvae, juveniles and adults. Most importantly, it has been associated with recurrent mortality outbreaks in several major bivalve-producing countries, including France, Spain, Chile and the USA [2,3]. *V. europaeus* outbreaks are associated with elevated seawater temperatures (18–25 °C), as occurs in hatcheries where water temperature is usually increased (>18 °C) to support larval cultures [4, 3].

The bacterial pangenome is defined as the entire gene repertoire of a microbial species [5]. Advances in pangenome studies have improved our understanding of bacterial genomic adaptations to the environment and contributed to the taxonomic and evolutionary research of microbial species [6,8]. In a previous study from our lab, the pangenome of *V. europaeus* was characterized, with 61% of the total genes belonging to the accessory genome [9]. This accessory genome consists of (i) genes that are present in most individuals (95–99%) of the studied clade (known as shell core), conceptually similar to the core genome but more suitable for large-scale genomic comparisons as it allows identification of missing genes due to evolutionary loss events or technical reasons, such as assembly or gene calling artefacts [10]; (ii) genes that are conserved between some individuals of the group (15–95%) but not most (known as soft core); and (iii) genes that are rare within the population and found only in one or a few individuals (0–15%) (known as cloud genome) [10,11]. In the case of *V. europaeus*, 4 and 70% of the accessory genes were assigned to the soft core and cloud genome, respectively, accounting for both fractions 45% of the total pangenome [9]. The accessory genome is composed principally of polymorphic strain-specific DNA segments named regions of genomic plasticity (RGPs), which are defined as clusters of specific genes located in a highly variable genomic region [10,12]. Most of the RGPs are embedded in insertion spots. These spots are more active in terms of acquisition rate of new elements than the rest of the genome and, in consequence, tend to have a much more diverse gene content, differentiating even between closely related individuals [10].

Most genes in the soft core and cloud fractions are thought to originate from horizontal gene transfer (HGT) events [10]. HGT is a major source of variability in prokaryotic genomes and enables efficient niche adaptation [13,14]. This process is mediated by three main mechanisms: (i) transformation, the uptake of external free DNA by bacteria; (ii) transduction, the transfer of DNA between bacterial hosts via phages; and (iii) conjugation, the direct transfer of DNA between adjacent bacteria via pili structures [15]. Most RGPs are typically associated with mobile genetic elements (MGEs) and are therefore acquired through HGT events [10].

MGEs are genetic material capable of intra- and intercellular mobility and constitute the microbial mobilome [16,17]. MGEs often harbour genes conferring selective advantages, such as virulence genes, antimicrobial resistance genes (ARGs) or anti-phage defence systems, which promote niche adaptation, bacterial evolution and dissemination of these traits [18,20]. Such characteristics act as a trade-off for the persistence of MGEs in bacterial genomes, since hosts tend to eliminate unnecessary genetic material to avoid metabolic burden [18,21,23]. MGEs include transposons, integrons, integrative and conjugative/mobilizable elements (ICEs/IMEs), microsatellites, prophages, phage satellites and plasmids, among others [24, 17,25]. In the case of plasmids, their mobility can be restricted, being classified as conjugative, mobilizable or non-mobilizable [26]. ICEs (10–700 kb) can excise from the host genome, transfer by conjugation and reintegrate into the host DNA, while IMEs (2–50 kb) depend on co-resident conjugative elements for transfer [27, 28, 29]. Lysogenic prophages integrate into the host genome or persist as plasmid prophages and can reactivate through the lytic cycle or degenerate into residual forms through gene inactivation [30]. Phage satellites act as parasites of bacterial phages, using their machinery to replicate and transfer horizontally between bacteria. Four families of phage satellites have been described: P4-like, phage-inducible chromosomal islands (PICIs), capsid-forming PICIs and PICI-like elements [24].

Furthermore, studies about the structure and characteristics of the accessory genome of non-clinical *Vibrio* spp. are scarce. Even though their accessory genome is a well-known reservoir of genetic material that drives bacterial adaptation, its functional impact and evolution remain largely unexplored. Here, we explore for the first time the structural organization of the accessory genome of the bivalve pathogen *V. europaeus* by comparative genomics, including non-chromosomic (plasmids) and chromosomic genetic elements (prophages, ICEs/IMEs, phage satellites and unclassified chromosomic RGPs), as well as the genes of interest encoded by those genetic elements, such as virulence, production of secondary metabolites, resistance to antimicrobials or anti-phage defence systems. This study aims to provide new insights into the role of the accessory genetic elements in the evolution, adaptation and diversity of the shellfish pathogen *V. europaeus*.

## Methods

### Identification of plasmids

A total of 39 *V*. *europaeus* assemblies were used for this study (Table 1). Genomes were previously sequenced in our laboratory, and the methodology is further described in Rodriguez *et al.* [9]. Briefly, two sequencing approaches were applied for genome assembly. Initially, bacterial genomes were sequenced using short-read technology (HiSeq4000 sequencer, Illumina) to generate contig-level assemblies [9] (Table 1). Subsequently, four representative strains – the type strain CECT8136 and isolates CECT8427, PP2-843 and EX1 (Table 1) – were subjected to long-read sequencing using either the PacBio Sequel II platform (PacBio) or the MinION sequencer with the Rapid Sequencing gDNA-barcoding kit (ONT) for the EX1 genome. Chromosome-level assemblies were then obtained and polished with Illumina reads from the short-read sequencing to produce high-quality assemblies [9]. In total, six chromosome-level assemblies were used in this study: CECT8136ᵀ, CECT8427, PP2-843 and EX1 (sequenced by Rodriguez *et al.* [9]), together with NPI-1 and CECT8426 retrieved from NCBI, although previously sequenced in our laboratory [3] (Table 1). These genome assemblies were used by Rodriguez *et al.* [9] to construct the *V. europaeus* pangenome using Roary [11]. The resulting pangenome comprised 9,860 genes, including 39% (3,846 genes) assigned to the core genome and 61% (6,014 genes) belonging to the accessory genome, of which 26, 4 and 70% corresponded to the shell, soft core and cloud gene fractions, respectively.

**Fig. 1.**
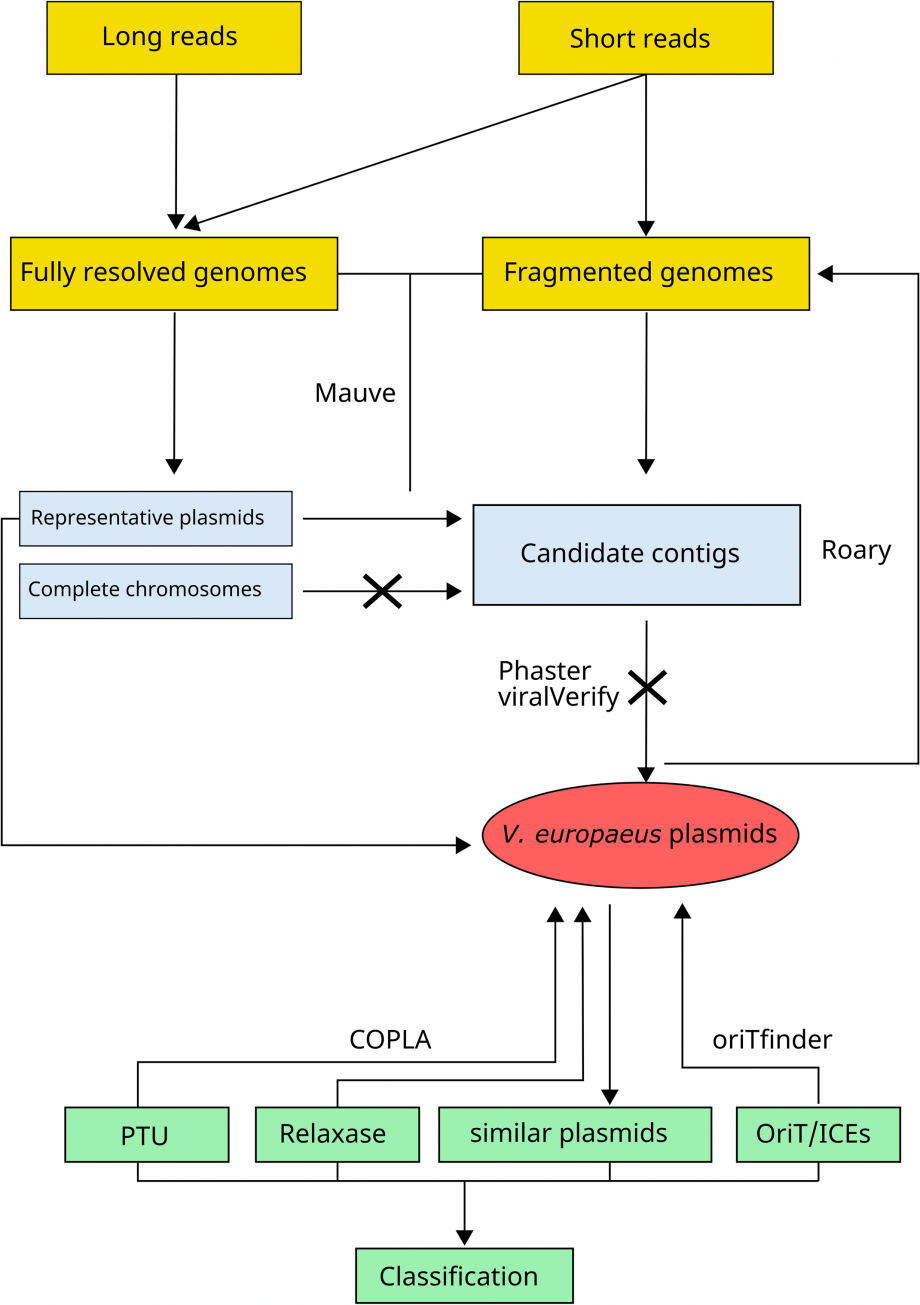
Bioinformatic workflow for plasmid identification and classification in *V. europaeus* genomes. Arrows with crosses represent genetic elements deleted in the final database. Software used in each step is also indicated.

Fully resolved genomes (CECT 8136, CECT 8427, CECT 8426, PP2-843, EX1 and NPI-1 strains) were assembled through long-read sequencing, yielding non-fragmented plasmids (hereinafter referred to as representative plasmids) (Table 1). An overview of the workflow is presented in Fig. 1. Plasmids from short-read assemblies were identified by aligning contigs against each fully resolved genome using Mauve 2.4.0 under default parameters (match seed weight 15) [31]. (i) Contigs aligned with representative plasmids were designated as candidate contigs (Fig. 1), and (ii) contigs not aligned with representative plasmids nor with the bacterial chromosomes were analysed using Phaster (https://phaster.ca/) [32] and viralVerify 1.1 (https://github.com/ablab/viralVerify); those were not considered as plasmids if any positive match was found (Fig. 1). Then, representative plasmids from long-read assemblies and candidate contigs from short-read assemblies were designated as *V. europaeus* plasmids, and thus, plasmid sequences were extracted from the bacterial genomes (Fig. 1). An additional search was performed to ensure the detection of plasmids, and homology between the *V. europaeus* plasmid genes and the genomes was assessed with Roary 3.13.0 [11].

*V. europaeus* plasmids were analysed with COPLA 1.0 under default parameters and NBCI (refseq_reference_genomes database) to find the closest relative plasmids [33]. In addition, *V. europaeus* plasmids were classified using (i) a plasmid taxonomic unit (PTU) and (ii) the relaxase gene using MOBscan [34]. Finally, OriTfinder and ICE finder 1.0 [35,36] were used to determine if plasmids were non-mobilizable, mobilizable or conjugative (Fig. 1).

For plasmid pVE1 relatives (pVE1-like), all plasmids identified from *V. europaeus* genomes were aligned with Mauve as described above, and the consensus sequence was obtained with Jalview [37]. This consensus sequence was used as a reference to compare all pVE1-like sequences using the GView server (https://server.gview.ca/).

### Identification and classification of chromosomic RGPs

**Fig. 2.**
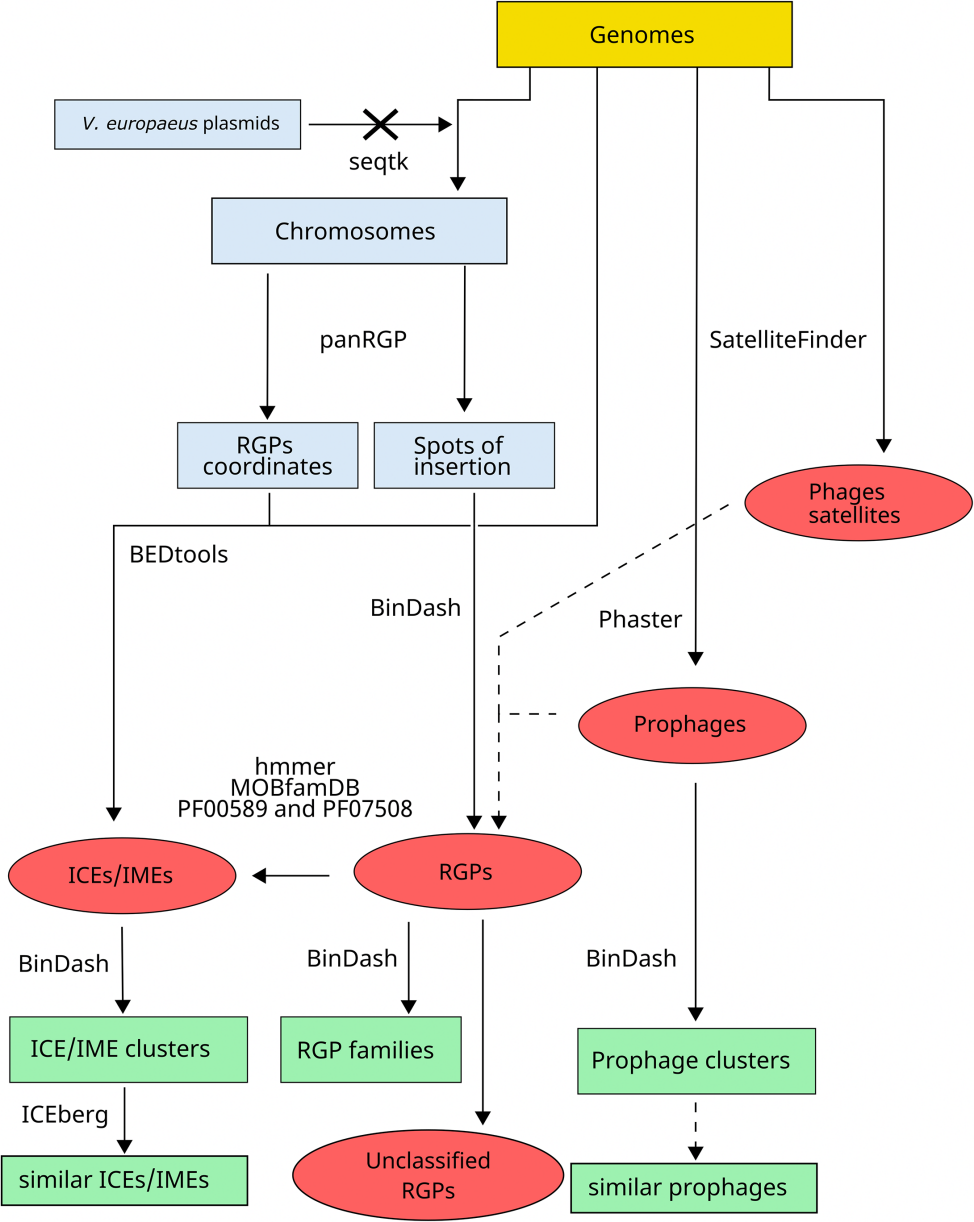
Bioinformatic workflow for identifying chromosomic genetic elements: ICEs/IMEs, prophages, phage satellites and unclassified chromosomic RGPs. Arrows with crosses represent genetic elements deleted in the final database. Comparative analyses that required a search of homologies with blast are indicated in dashed lines.

*V. europaeus* plasmids (Fig. 1) were removed from the genome assemblies using seqtk (https://github.com/lh3/seqtk). An overview of the workflow is presented in Fig. 2. The resulting bacterial chromosomes were annotated with Prokka 1.14.4 [38] and gff3 files were used to identify the chromosomic RGPs using panRGP under default arguments [10]. The genomic coordinates obtained with panRGP were used to extract the RGP sequences from the *V. europaeus* chromosome sequences using the getfasta utility from BEDtools 2.31.1 [39].

Chromosomic RGPs included all RGPs identified in the bacterial chromosomes, including those RGPs associated with any chromosomic MGEs, such as ICEs/IMEs, prophages and phage satellites. Thus, chromosomic RGPs identified in ICEs/IMEs, prophages and phage satellites were removed as described in the next section to obtain a final curated dataset designated as unclassified chromosomic RGPs. To evaluate the RGPs’ diversity, the mutation distance and the Jaccard Index of each pair of RGPs were calculated using BinDash 1.0 under default arguments [40]. Pairs of RGPs with a Jaccard Index value higher than the arithmetic mean were used to construct a network of RGPs using Cytoscape 3.10.0 [41]. The relationship between RGPs not assigned to a spot of insertion (hereafter designated as decontextualized RGPs) and the fragmentation of the genome was studied by calculating the Kendall rank correlation coefficient between the number of fragments of the *V. europaeus* genomes (contigs or scaffolds) and the proportion of decontextualized RGPs using R.

### Identification of ICEs/IMEs, prophages and phage satellites

Other MGEs, such as ICEs/IMEs, prophages and phage satellites, were identified from the bacterial chromosomes (Fig. 2).

1. ICEs/IMEs: RGP proteome obtained with Prokka was examined with hmmer 3.4 (http://hmmer.org/) to search for (i) relaxases using a curated relaxase profile HMM database (MOBfamDB [34]); and (ii) integrases and recombinases using the phage integrase family PF00589 (comprising 264,000 proteins) and the PF07508 domain, which is commonly found in putative integrases/recombinases of MGEs from diverse bacteria and phages (Fig. 2). Chromosomic RGPs with homologs for relaxase and integrase/recombinase were considered as ICEs/IMEs and thus those nucleotide sequences were removed from the chromosomic RGP. ICEs/IMEs were clustered by calculating the Jaccard Index using BinDash 1.0 [40] with a cutoff similarity value of 0.95, and ICE/IME clusters were plotted in a heatmap using the ComplexHeatmap 2.11.1 R package [42]. The distribution of the different ICE/IME clusters (clusters composed of more than one ICE/IME) within bacterial hosts was shown by the construction of a hierarchical edge bundling plot using the ggraph 2.1.0 R package (https://ggraph.data-imaginist.com) combined with the phylogenetic tree based on the core genome of *V. europaeus*, previously obtained by Rodriguez *et al.* [9]. Additionally, the Multigeneblast comparison method available in the ICEberg 3.0 database (https://tool2-mml.sjtu.edu.cn/ICEberg3) was used to search similar ICEs/IMEs (Fig. 2).
2. Prophages: prophages were identified by Phaster [32], and only ‘intact’ matches (high number of prophage CDS and presence of phage-related genes) were considered as prophages. Those prophage nucleotide sequences were blasted against the chromosomic RGP dataset using megablast default arguments [43]; if any match was found, those were removed from the chromosomic RGP dataset (Fig. 2). To study the similarity among *V. europaeus* prophages, a pairwise comparison was performed by using the Jaccard Index as described above for ICEs/IMEs. Finally, the nucleotide sequences of those prophages were queried in the nt NCBI database using megablast to look for similar prophages [43] (Fig. 2).
3. Phage satellites: detection of phage satellites was carried out using SatelliteFinder (https://galaxy.pasteur.fr/root?tool_id=toolshed.pasteur.fr/repos/fmareuil/satellitefinder/Satellite Finder/0.9) [44] and removed from the chromosomic RGP dataset to finally obtain the unclassified RGP dataset (Fig. 2).

### Annotation of genes of interest encoded in plasmids, ICEs/IMEs, prophages, phage satellites and unclassified chromosomic RGPs

Nucleotide sequences of genes identified from plasmids, ICEs/IMEs, prophages, phage satellites and unclassified chromosomic RGPs were used to construct a database with the makeblastdb application of blast+ using default arguments [43]. Then, genes related to virulence, antibiotic resistance, anti-phage defence systems and biosynthetic gene clusters (BGCs) identified previously by Rodriguez *et al.* [9] were blasted (blastn) against our database using megablast using default parameters (Word Size 28, Match Reward 1, Mismatch Penalty −2, Gap Open 0, Gap Extend 2.5, Gap Cost Type Linear, E-value 10) [43]. For unclassified chromosomic RGPs, results were manually marked in the networks of RGPs obtained previously, summarized and plotted as a bubble plot with the ggplot2 3.3.6 R package [45].

In our previous study, Rodriguez *et al.* [9] revealed a diverse repertoire of Tipe VI secretion system (T6SSs) in the *V. europaeus* pangenome, comprising three different T6SSs (T6SS1, T6SS2 and T6SS3). Among these, T6SS1 and T6SS3 belong to the core genome and are encoded on Chromosome 1. In contrast, T6SS2 was assigned to the accessory genome and, as we demonstrate in this study, is encoded on pVE1-related plasmids. T6SS3 differs from T6SS1 and T6SS2 and was classified as a T6SS i1 subtype. In contrast, both T6SS1 and T6SS2 showed a similar synteny and belong to the T6SS i5 subtype, although they exhibit significant differences among gene homologs. T6SS2 encoded by the pVE1-related plasmids was further characterized in this study using SecReT6 3.0 [46] and the CECT8136 genome as a reference. A phylogenetic analysis of the T6SS i5 subtype was performed: (i) as mentioned above, T6SS1 and T6SS2 belong to the T6SS i5 subtype and were therefore included in the phylogenetic analyses (T6SS3 was not included because it was assigned to the T6SS i1 subtype). For this purpose, the nucleotide sequences of T6SS1 and T6SS2 were extracted from the CECT8136 genome; (ii) NCBI accession numbers and genomic coordinates of all available T6SS type i5 were obtained from the SecReT6 3.0 database [46] and downloaded from NCBI using the getfasta utility from BEDtools 2.31.1 [39]; and (iii) the sequences of the T6SS structural gene *tssB* were used to construct the T6SS type i5 phylogeny using CORASON under default parameter (https://github.com/nselem/corason). The resulting Newick tree was visualized in iTOL [47]. The Scalable Vector Graphics file containing the phylogenetic tree with the T6SS synteny was manually modified to show the minimal clade containing the Chr1-T6SS (T6SS1) and the plasmid-T6SS (T6SS2) subtype i5 of *V. europaeus*.

### Distribution of accessory genetic elements among the pangenome accessory fractions

Genes encoded by plasmids, ICEs/IMEs, prophages, phage satellites and unclassified chromosomic RGPs were assigned to specific fractions of the accessory genome (soft core, shell genes and cloud fractions) as follows: (i) the accessory genes associated with soft core, shell and cloud fractions were obtained from the *V. europaeus* pangenome using Roary 3.13.0 [11]; (ii) the nucleotide sequences of plasmids, ICEs/IMEs, prophages, phage satellites and unclassified chromosomic RGPs were compiled into a multi-FASTA file and annotated with Prokka [38], obtaining an ffn file containing the protein-coding sequences. These sequences were then blasted against the soft core, shell or cloud fractions using megablast with default arguments [43]. Gene ID of perfect matches was assigned to an accessory genome fraction using the gene_presence_absence.csv file generated by Roary [11] and an ad hoc Python script (https://github.com/sergio-c-r/gene-freq-from-roary/blob/main/converge).

## Results

### Novel pVE1-like plasmids represent the most widespread MGEs, encoding the highest number of virulence genes, BGCs and a high diversity of anti-phage defence systems

The *V. europaeus* plasmidome was composed of a total of 52 plasmids. Comparative analysis classified them into five different groups (Table 2): (i) pVE1-like plasmids, harboured by most *V. europaeus* strains (38/39), ranging in size from 308.9 to 419.27 kb; (ii) pVE2-like plasmids, present in eight strains (64.02–82.13 kb); (iii) pVE3-like plasmids, found in four strains (168.72–168.83 kb); (iv) pVE4, detected in two strains (41.65 kb); and (v) pVE5, present only in a single strain (71.05 kb). No PTU was identified by COPLA; consequently, any plasmid could not be taxonomically classified by this method. However, closely related plasmids matched in databases (Table 2): (i) p251 (NZ_CP009356.1; *Vibrio tubiashii* ATCC 19109) to pVE1-like; (ii) p57 (NZ_CP009358.1; *V. tubiashii* ATCC 19109) to pVE2-like; and (iii) p1 (CP092386.1; *Vibrio gigantis* ACE001) to pVE3-like plasmids. No related plasmids were identified for pVE4 and pVE5. Plasmid classification based on relaxase families revealed that pVE1-like and pVE2-like plasmids contained a relaxase from the MOBF family, while pVE3-like and pVE4 plasmids harboured relaxases from the MOBH and MOBC families, respectively (Table 2). Interestingly, a second relaxase belonging to the MOBC family was identified in the pVE1 plasmids of the French strains 07/121 T1, 07/112 T1 and 07/110 T1 (Table 2).

**Table 2.** Plasmids identified from the *V. europaeus* genomes Plasmid classification based on the relaxase genes is indicated. Integrases and T4CP and MPF proteins were also identified.

Strain	Plasmid type	Size (kb)	Relaxase	T4CP	MPF (T4SS)	Integrase	Closest relatives
EX1	pVE1	330.1	MOBF	TrwB_AAD_bind, FtsK_SpoIIIE, TraD_N	TraG_F, TraH_F, TraH_F, TraB_F, TraF_F, TraN_F, TraN_F, TrbC_F, TraU_F, TraC_F, TraC_F, TraC_F, TraC_F, TraC_F, TraB_F, TraK_F, TraE_F, TraE_F, TraL_F, TraA_F	Phage_integrase, rve	*V. tubiashii* ATCC 19109 p251 (NZ_CP009356.1)
2895	pVE1	329.0	MOBF	TrwB_AAD_bind	TraA, F_traL, TraE, TraK, F_traB, F_traV, virb4, F_traW, F_traU, F_trbC, F_traN, TraF, F_traF, F_traH, F_traG	Phage_integrase, rve	
2909	pVE1	386.1	MOBF	TrwB_AAD_bind (2)	TraA, F_traL, TraE, TraK, F_traB, F_traV, virb4, F_traW, F_traU, F_trbC, F_traN, TraF, F_traF, F_traH, F_traG	Phage_integrase	
2930	pVE1	329.3	MOBF	TrwB_AAD_bind	TraA, F_traL, TraE, TraK, F_traB, F_traV, virb4, F_traW, F_traU, F_trbC, F_traN, TraF, F_traF, F_traH, F_traG	Phage_integrase, rve	
2945	pVE1	329.0	MOBF	TrwB_AAD_bind	TraA, F_traL, TraE, TraK, F_traB, F_traV, virb4, F_traW, F_traU, F_trbC, F_traN, TraF, F_traF, F_traH, F_traG	Phage_integrase, rve	
2951	pVE1	329.0	MOBF	TrwB_AAD_bind	TraA, F_traL, TraE, TraK, F_traB, F_traV, virb4, F_traW, F_traU, F_trbC, F_traN, TraF, F_traF, F_traH, F_traG	Phage_integrase, rve	
2967	pVE1	386.1	MOBF	TrwB_AAD_bind (2)	TraA, F_traL, TraE, TraK, F_traB, F_traV, virb4, F_traW, F_traU, F_trbC, F_traN, TraF, F_traF, F_traH, F_traG	Phage_integrase	
2968	pVE1	329.0	MOBF	TrwB_AAD_bind	TraA, F_traL, TraE, TraK, F_traB, F_traV, virb4, F_traW, F_traU, F_trbC, F_traN, TraF, F_traF, F_traH, F_traG	Phage_integrase, rve	
2969	pVE1	329.0	MOBF	TrwB_AAD_bind	TraA, F_traL, TraE, TraK, F_traB, F_traV, virb4, F_traW, F_traU, F_trbC, F_traN, TraF, F_traF, F_traH, F_traG	Phage_integrase, rve	
2971	pVE1	329.0	MOBF	TrwB_AAD_bind	TraA, F_traL, TraE, TraK, F_traB, F_traV, virb4, F_traW, F_traU, F_trbC, F_traN, TraF, F_traF, F_traH, F_traG,	Phage_integrase, rve	
2974	pVE1	386.1	MOBF	TrwB_AAD_bind (2)	TraA, F_traL, TraE, TraK, F_traB, F_traV, virb4, F_traW, F_traU, F_trbC, F_traN, TraF, F_traF, F_traH, F_traG	Phage_integrase	
2975	pVE1	329.0	MOBF	TrwB_AAD_bind	TraA, F_traL, TraE, TraK, F_traB, F_traV, virb4, F_traW, F_traU, F_trbC, F_traN, TraF, F_traF, F_traH, F_traG	Phage_integrase, rve	
3454	pVE1	330.0	MOBF	TrwB_AAD_bind	TraA, F_traL, TraE, TraK, F_traB, F_traV, virb4, F_traW, F_traU, F_trbC, F_traN, TraF, F_traF, F_traH, F_traG	Phage_integrase, rve	
3492	pVE1	329.4	MOBF	TrwB_AAD_bind	TraA, F_traL, TraE, TraK, F_traB, F_traV, virb4, F_traW, F_traU, F_trbC, F_traN, TraF, F_traF, F_traH, F_traG	Phage_integrase, rve(2)	
3610	pVE1	329.1	MOBF	TrwB_AAD_bind	TraA, F_traL, TraE, TraK, F_traB, F_traV, virb4, F_traW, F_traU, F_trbC, F_traN, TraF, F_traF, F_traH, F_traG	Phage_integrase, rve	
2909	pVE1	386.1	MOBF	TrwB_AAD_bind (2)	TraA, F_traL, TraE, TraK, F_traB, F_traV, virb4, F_traW, F_traU, F_trbC, F_traN, TraF, F_traF, F_traH, F_traG	Phage_integrase	
2930	pVE1	329.3	MOBF	TrwB_AAD_bind	TraA, F_traL, TraE, TraK, F_traB, F_traV, virb4, F_traW, F_traU, F_trbC, F_traN, TraF, F_traF, F_traH, F_traG	Phage_integrase, rve	
2945	pVE1	329.0	MOBF	TrwB_AAD_bind	TraA, F_traL, TraE, TraK, F_traB, F_traV, virb4, F_traW, F_traU, F_trbC, F_traN, TraF, F_traF, F_traH, F_traG	Phage_integrase, rve	
2951	pVE1	329.0	MOBF	TrwB_AAD_bind	TraA, F_traL, TraE, TraK, F_traB, F_traV, virb4, F_traW, F_traU, F_trbC, F_traN, TraF, F_traF, F_traH, F_traG	Phage_integrase, rve	
2967	pVE1	386.1	MOBF	TrwB_AAD_bind (2)	TraA, F_traL, TraE, TraK, F_traB, F_traV, virb4, F_traW, F_traU, F_trbC, F_traN, TraF, F_traF, F_traH, F_traG	Phage_integrase	
2968	pVE1	329.0	MOBF	TrwB_AAD_bind	TraA, F_traL, TraE, TraK, F_traB, F_traV, virb4, F_traW, F_traU, F_trbC, F_traN, TraF, F_traF, F_traH, F_traG	Phage_integrase, rve	
2969	pVE1	329.0	MOBF	TrwB_AAD_bind	TraA, F_traL, TraE, TraK, F_traB, F_traV, virb4, F_traW, F_traU, F_trbC, F_traN, TraF, F_traF, F_traH, F_traG	Phage_integrase, rve	
2971	pVE1	329.0	MOBF	TrwB_AAD_bind	TraA, F_traL, TraE, TraK, F_traB, F_traV, virb4, F_traW, F_traU, F_trbC, F_traN, TraF, F_traF, F_traH, F_traG,	Phage_integrase, rve	
2974	pVE1	386.1	MOBF	TrwB_AAD_bind (2)	TraA, F_traL, TraE, TraK, F_traB, F_traV, virb4, F_traW, F_traU, F_trbC, F_traN, TraF, F_traF, F_traH, F_traG	Phage_integrase	
2975	pVE1	329.0	MOBF	TrwB_AAD_bind	TraA, F_traL, TraE, TraK, F_traB, F_traV, virb4, F_traW, F_traU, F_trbC, F_traN, TraF, F_traF, F_traH, F_traG	Phage_integrase, rve	
3454	pVE1	330.0	MOBF	TrwB_AAD_bind	TraA, F_traL, TraE, TraK, F_traB, F_traV, virb4, F_traW, F_traU, F_trbC, F_traN, TraF, F_traF, F_traH, F_traG	Phage_integrase, rve	
3492	pVE1	329.4	MOBF	TrwB_AAD_bind	TraA, F_traL, TraE, TraK, F_traB, F_traV, virb4, F_traW, F_traU, F_trbC, F_traN, TraF, F_traF, F_traH, F_traG	Phage_integrase, rve(2)	
3610	pVE1	329.1	MOBF	TrwB_AAD_bind	TraA, F_traL, TraE, TraK, F_traB, F_traV, virb4, F_traW, F_traU, F_trbC, F_traN, TraF, F_traF, F_traH, F_traG	Phage_integrase, rve	
3614	pVE1	329.0	MOBF	TrwB_AAD_bind	TraA, F_traL, TraE, TraK, F_traB, F_traV, virb4, F_traW, F_traU, F_trbC, F_traN, TraF, F_traF, F_traH, F_traG	Phage_integrase, rve	
L2	pVE1	370.2	MOBF	TrwB_AAD_bind (2)	TraA, F_traL, TraE, TraK, F_traB, F_traV, virb4, F_traW, F_traU, F_trbC, F_traN, TraF, F_traF, F_traH, F_traG	Phage_integrase	
L20	pVE1	326.8	MOBF	TrwB_AAD_bind	TraA, F_traL, TraE, TraK, F_traB, F_traV, virb4, F_traW, F_traU, F_trbC, F_traN, TraF, F_traF, F_traH, F_traG	Phage_integrase	
L3	pVE1	326.6	MOBF	TrwB_AAD_bind	TraA, F_traL, TraE, TraK, F_traB, F_traV, virb4, F_traW, F_traU, F_trbC, F_traN, TraF, F_traF, F_traH, F_traG	Phage_integrase	
L4	pVE1	326.5	MOBF	TrwB_AAD_bind	TraA, F_traL, TraE, TraK, F_traB, F_traV, virb4, F_traW, F_traU, F_trbC, F_traN, TraF, F_traF, F_traH, F_traG	Phage_integrase	
PP-635	pVE1	326.4	MOBF	TrwB_AAD_bind	TraA, F_traL, TraE, TraK, F_traB, F_traV, virb4, F_traW, F_traU, F_trbC, F_traN, TraF, F_traF, F_traH, F_traG	Phage_integrase	
PP-654	pVE1	326.4	MOBF	TrwB_AAD_bind	TraA, F_traL, TraE, TraK, F_traB, F_traV, virb4, F_traW, F_traU, F_trbC, F_traN, TraF, F_traF, F_traH, F_traG	Phage_integrase	
PP-660	pVE1	326.5	MOBF	TrwB_AAD_bind	TraA, F_traL, TraE, TraK, F_traB, F_traV, virb4, F_traW, F_traU, F_trbC, F_traN, TraF, F_traF, F_traH, F_traG	Phage_integrase	
CECT8136	pVE1	327.2	MOBF	TrwB_AAD_bind	TraA, F_traL, TraE, TraK, F_traB, F_traV, virb4, F_traW, F_traU, F_trbC, F_traN, TraF, F_traF, F_traH, F_traG	Phage_integrase	
PP2-843	pVE1	389.5	MOBF	TrwB_AAD_bind (2)	TraA, F_traL, TraE, TraK, F_traB, F_traV, virb4, F_traW, F_traU, F_trbC, F_traN, TraF, F_traF, F_traH, F_traG	Phage_integrase	
PP2-978	pVE1	385.6	MOBF	TrwB_AAD_bind (2)	TraA, F_traL, TraE, TraK, F_traB, F_traV, virb4, F_traW, F_traU, F_trbC, F_traN, TraF, F_traF, F_traH, F_traG	Phage_integrase	
NPI-1	pVE1	335.7	MOBF	–	–	–	
071316 f	pVE1	309.0	MOBF	TrwB_AAD_bind	TraA, F_traL, TraE, TraK, F_traB, F_traV, TraC_F_IV, virb4, F_traW, F_traU, F_trbC, F_traN, TraF, F_traF, F_traH, F_traG	Phage_integrase	
CECT8427	pVE1	362.6	MOBF	TrwB_AAD_bind	F_traL, TraE, TraK, F_traB, F_traV, virb4, F_traW, F_traU, F_trbC, F_traN, TraF, F_traF, F_traH, F_traG	Phage_integrase(3), rve	
07/38_2 T2	pVE1	393.6	MOBF	TrwB_AAD_bind	TraA, F_traL, TraE, TraK, F_traB, F_traV, virb4, F_traW, F_traU, F_trbC, F_traN, TraF, F_traF, F_traH, F_traG	Phage_integrase	
07/108_T1	pVE1	309.0	MOBF	–	–	Phage_integrase	
07/110_T1	pVE1	395.0	MOBF,MOBC	TrwB_AAD_bind	TraA, F_traL, TraE, TraK, F_traB, F_traV, virb4, F_traW, F_traU, F_trbC, F_traN, TraF, F_traF, F_traH, F_traG	Phage_integrase(3)	
07/112_T1	pVE1	395.0	MOBF,MOBC	TrwB_AAD_bind	TraA, F_traL, TraE, TraK, F_traB, F_traV, virb4, F_traW, F_traU, F_trbC, F_traN, TraF, F_traF, F_traH, F_traG	Phage_integrase(3)	
07/116_T1	pVE1	373.1	MOBF	TrwB_AAD_bind	TraA, F_traL, TraE, TraK, F_traB, F_traV, virb4, F_traW, F_traU, F_trbC, F_traN, TraF, F_traF, F_traH, F_traG	Phage_integrase(3), TIGR02224	
07/117_T1	pVE1	373.2	MOBF	TrwB_AAD_bind (2)	TraA, F_traL, TraE, TraK, F_traB, F_traV, virb4, F_traW, F_traU, F_trbC, F_traN, TraF, F_traF, F_traH, F_traG	Phage_integrase(3), TIGR02224	
3614	pVE1	329.0	MOBF	TrwB_AAD_bind	TraA, F_traL, TraE, TraK, F_traB, F_traV, virb4, F_traW, F_traU, F_trbC, F_traN, TraF, F_traF, F_traH, F_traG	Phage_integrase, rve	
L2	pVE1	370.2	MOBF	TrwB_AAD_bind (2)	TraA, F_traL, TraE, TraK, F_traB, F_traV, virb4, F_traW, F_traU, F_trbC, F_traN, TraF, F_traF, F_traH, F_traG	Phage_integrase	
L20	pVE1	326.8	MOBF	TrwB_AAD_bind	TraA, F_traL, TraE, TraK, F_traB, F_traV, virb4, F_traW, F_traU, F_trbC, F_traN, TraF, F_traF, F_traH, F_traG	Phage_integrase	
L3	pVE1	326.6	MOBF	TrwB_AAD_bind	TraA, F_traL, TraE, TraK, F_traB, F_traV, virb4, F_traW, F_traU, F_trbC, F_traN, TraF, F_traF, F_traH, F_traG	Phage_integrase	
L4	pVE1	326.5	MOBF	TrwB_AAD_bind	TraA, F_traL, TraE, TraK, F_traB, F_traV, virb4, F_traW, F_traU, F_trbC, F_traN, TraF, F_traF, F_traH, F_traG	Phage_integrase	
PP-635	pVE1	326.4	MOBF	TrwB_AAD_bind	TraA, F_traL, TraE, TraK, F_traB, F_traV, virb4, F_traW, F_traU, F_trbC, F_traN, TraF, F_traF, F_traH, F_traG	Phage_integrase	
PP-654	pVE1	326.4	MOBF	TrwB_AAD_bind	TraA, F_traL, TraE, TraK, F_traB, F_traV, virb4, F_traW, F_traU, F_trbC, F_traN, TraF, F_traF, F_traH, F_traG	Phage_integrase	
PP-660	pVE1	326.5	MOBF	TrwB_AAD_bind	TraA, F_traL, TraE, TraK, F_traB, F_traV, virb4, F_traW, F_traU, F_trbC, F_traN, TraF, F_traF, F_traH, F_traG	Phage_integrase	
CECT8136	pVE1	327.2	MOBF	TrwB_AAD_bind	TraA, F_traL, TraE, TraK, F_traB, F_traV, virb4, F_traW, F_traU, F_trbC, F_traN, TraF, F_traF, F_traH, F_traG	Phage_integrase	
PP2-843	pVE1	389.5	MOBF	TrwB_AAD_bind (2)	TraA, F_traL, TraE, TraK, F_traB, F_traV, virb4, F_traW, F_traU, F_trbC, F_traN, TraF, F_traF, F_traH, F_traG	Phage_integrase	
PP2-978	pVE1	385.6	MOBF	TrwB_AAD_bind (2)	TraA, F_traL, TraE, TraK, F_traB, F_traV, virb4, F_traW, F_traU, F_trbC, F_traN, TraF, F_traF, F_traH, F_traG	Phage_integrase	
NPI-1	pVE1	335.7	MOBF	–	–	–	
071316 f	pVE1	309.0	MOBF	TrwB_AAD_bind	TraA, F_traL, TraE, TraK, F_traB, F_traV, TraC_F_IV, virb4, F_traW, F_traU, F_trbC, F_traN, TraF, F_traF, F_traH, F_traG	Phage_integrase	
CECT8427	pVE1	362.6	MOBF	TrwB_AAD_bind	F_traL, TraE, TraK, F_traB, F_traV, virb4, F_traW, F_traU, F_trbC, F_traN, TraF, F_traF, F_traH, F_traG	Phage_integrase(3), rve	
07/38_2 T2	pVE1	393.6	MOBF	TrwB_AAD_bind	TraA, F_traL, TraE, TraK, F_traB, F_traV, virb4, F_traW, F_traU, F_trbC, F_traN, TraF, F_traF, F_traH, F_traG	Phage_integrase	
07/108_T1	pVE1	309.0	MOBF	–	–	Phage_integrase	
07/110_T1	pVE1	395.0	MOBF,MOBC	TrwB_AAD_bind	TraA, F_traL, TraE, TraK, F_traB, F_traV, virb4, F_traW, F_traU, F_trbC, F_traN, TraF, F_traF, F_traH, F_traG	Phage_integrase(3)	
07/112_T1	pVE1	395.0	MOBF,MOBC	TrwB_AAD_bind	TraA, F_traL, TraE, TraK, F_traB, F_traV, virb4, F_traW, F_traU, F_trbC, F_traN, TraF, F_traF, F_traH, F_traG	Phage_integrase(3)	
07/116_T1	pVE1	373.1	MOBF	TrwB_AAD_bind	TraA, F_traL, TraE, TraK, F_traB, F_traV, virb4, F_traW, F_traU, F_trbC, F_traN, TraF, F_traF, F_traH, F_traG	Phage_integrase(3), TIGR02224	
07/117_T1	pVE1	373.2	MOBF	TrwB_AAD_bind (2)	TraA, F_traL, TraE, TraK, F_traB, F_traV, virb4, F_traW, F_traU, F_trbC, F_traN, TraF, F_traF, F_traH, F_traG	Phage_integrase(3), TIGR02224	
8426	pVE1	419.3	MOBF	TrwB_AAD_bind	TraA, F_traL, TraE, TraK, F_traB, F_traV, virb4, F_traW, F_traU, F_trbC, F_traN, TraF, F_traF, F_traH, F_traG	Phage_integrase	
07/120_T1	pVE1	415.2	MOBF	TrwB_AAD_bind	TraA, F_traL, TraE, TraK, F_traB, F_traV, virb4, F_traW, F_traU, F_trbC, F_traN, TraF, F_traF, F_traH, F_traG	Phage_integrase	
07/121_1 T1	pVE1	395.0	MOBF,MOBC	TrwB_AAD_bind	TraA, F_traL, TraE, TraK, F_traB, F_traV, virb4(2), F_traW, F_traU, F_trbC, F_traN, TraF, F_traF, F_traH, F_traG, FATA_trsC	Phage_integrase(3)	
CECT8136	pVE2	64.1	MOBF	TrwB_AAD_bind	Orf169_F, TraD_F, TraG_F, TraH_F, TraB_F, TraF_F, TraN_F, TrbC_F, TraU_F, TraW_F, TraC_F, TraC_F, TraV, TraB_F, TraK_F, TraE_F, TraL_F, TraA_F	Phage_integrase	V. tubiashii ATCC 19109 p57 (NZ_CP009358.1)
2930	pVE2	64.2	MOBF	TrwB_AAD_bind	Orf169_F, TraD_F, TraG_F, TraH_F, TraB_F, TraF_F, TraN_F, TrbC_F, TraU_F, TraW_F, TraC_F, TraC_F, TraV, TraB_F, TraK_F, TraE_F, TraL_F, TraA_F	Phage_integrase	
3454	pVE2	65.0	MOBF	TrwB_AAD_bind	TraF_F, TraN_F, TrbC_F, TraU_F, TraW_F, TraC_F, TraV, TraB_F, TraK_F, TraE_F, TraL_F, TraA_F	Phage_integrase	
3492	pVE2	64.3	MOBF	TrwB_AAD_bind	Orf169_F, TraD_F, TraG_F, TraH_F, TraB_F, TraF_F, TraN_F, TrbC_F, TraU_F, TraW_F, TraC_F, TraC_F, TraV, TraB_F, TraK_F, TraE_F	Phage_integrase	
3610	pVE2	64.2	MOBF	TrwB_AAD_bind	Orf169_F, TraD_F, TraG_F, TraH_F, TraB_F, TraF_F, TraN_F, TrbC_F, TraU_F, TraW_F, TraC_F, TraC_F, TraV, TraB_F, TraK_F, TraE_F	Phage_integrase	
L20	pVE2	64.0	MOBF	TrwB_AAD_bind	TraA_F, TraL_F, TraE_F, TraK_F, TraB_F, TraV, TraC_F, TraW_F, TraU_F, TrbC_F, TraN_F, TraF_F, TraB_F, TraH_F, TraG_F	Phage_integrase	
L3	pVE2	64.2	MOBF	TrwB_AAD_bind	Orf169_F, TraD_F, TraG_F, TraH_F, TraB_F, TraF_F, TraN_F, TrbC_F, TraU_F, TraW_F, TraC_F, TraC_F, TraV, TraB_F, TraK_F, TraE_F	Phage_integrase	
07/115 T2	pVE2	82.1	MOBF	TrwB_AAD_bind	TraA, F_traL, TraE, TraK, F_traB, F_traV, virb4, F_traW, F_traU, F_trbC, F_traN, TraF, F_traF, F_traH, F_traG, T_virB1	Phage_integrase, rve	
3454	pVE3	168.7	MOBH	t4cp2	TraF_F, TraH_F, TraG_F	Phage_integrase	V. gigantis ACE001 plasmid (NZ_CP092386.1)
3492	pVE3	168.7	MOBH	t4cp2	TraF_F, TraH_F, TraG_F	Phage_integrase	
3610	pVE3	168.8	MOBH	t4cp2	TraF_F, TraH_F, TraG_F	Phage_integrase	
3614	pVE3	168.8	MOBH	t4cp2	TraG_F, TraH_F, TraF_F	Phage_integrase	
L3	pVE4	41.7	MOBC	–	VirD4, VirB11, VirB1, TraJ_I, Tfc2, VirB10, VirB9, VirB8, VirB6, VirB5, VirB4, VirB2	–	–
L20	pVE4	41.7	MOBC	–	VirD4, VirB11, VirB1, TraJ_I, Tfc2, VirB10, VirB9, VirB8, VirB6, VirB5, VirB4, VirB2	–	–
CECT8427	pVE5	71.05	–	t4cp2	Tfc19,Tfc22,Tfc23,Tfc24,Tfc4,TraJ_I,Tfc16, Tfc15,Tfc14,Tfc13,Tfc12,Tfc11,Tfc11,Tfc9, Tfc8,Tfc5,Tfc4,Tfc3,Tfc2	Phage_integrase	–

**Fig. 3.**
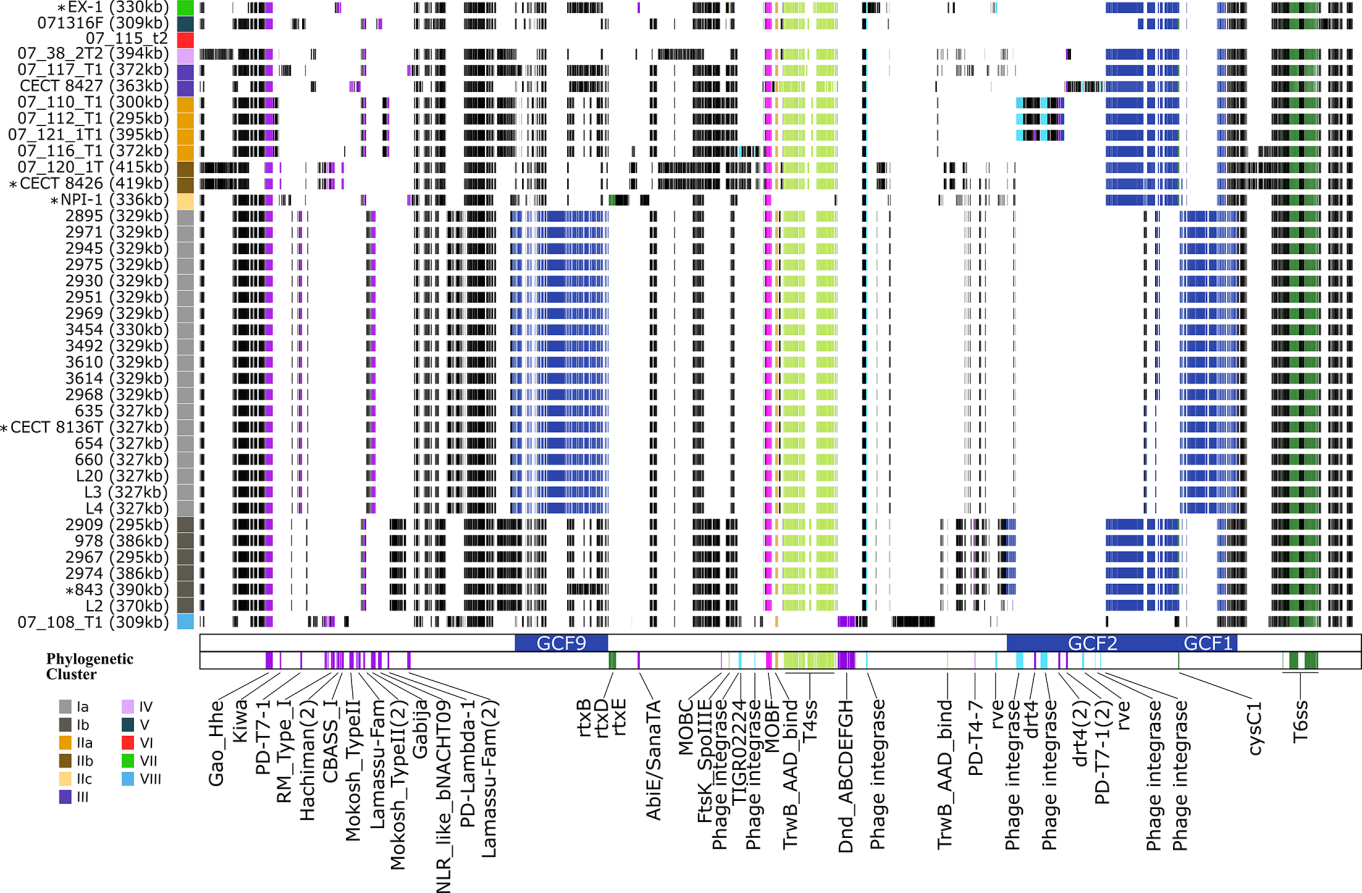
Genetic structure of pVE1-like plasmids. Phylogenetic clusters (I–VIII) based on the core genome [9] are depicted in colours. Plasmids identified from fully resolved assemblies are marked with asterisks. Plasmid sizes are shown in brackets. Genes were highlighted with different colours: defence systems (purple), BGCs (dark blue), virulence genes (dark blue), T6SS2 (green), relaxase (pink), T4CP (orange), MPF (T4SS) (light green) and integrases (light blue).

According to their mobility, most pVE1-like plasmids, as well as all pVE2-like and pVE3-like plasmids, were defined as conjugative plasmids, even though no known *oriT* sequence was identified. This classification was based on the presence of the necessary conjugation machinery, including a relaxase, a type IV coupling protein (T4CP) and a mating pair formation (MPF) complex encoded by T4SS genes, which facilitate the assembly and functionality of the mating channel. In contrast, mobilizable plasmids lack at least the MPF and rely on the MPF of another genetic element present in the cell. Examples of mobilizable plasmids were found among pVE1-like plasmids: strains 07/108 T1 and NPI-1 harboured mobilizable pVE1-like plasmids that contained only the relaxase gene (Table 2 and Fig. 3). In contrast, pVE4 and pVE5 were classified as mobilizable and potentially mobilizable, respectively. Specifically, pVE4 lacked T4CP genes, whereas pVE5 encoded T4SS and T4CP genes but no known relaxase (Table 2).

This report constituted the first description of the novel pVE1-like plasmids. Indisputably, pVE1-like plasmids were the largest (>300 kb; Table 2) and most abundant plasmids in the *V. europaeus* plasmidome: 97.4% of the strains harboured this plasmid, including Spanish, French, American and Chilean strains (with the exception of the French strain 07/115 T1) (Table 2 and Fig. 3). This plasmid was the only one present in 29 of the 39 strains, while the remaining 9 strains carried the pVE1-like plasmid together with additional plasmid(s). These strains harboured, in combination with pVE1, either two plasmids (pVE1+pVE2: strains CECT8136 and 2930; pVE1+pVE3: strain 3614; pVE1+pVE1: strain CECT8427) or up to three plasmids (pVE1+pVE2+pVE3: strains 3610, 3454 and 3492; pVE1+pVE2+pVE4: strains L3 and L20) (Table 2).

**Fig. 4.**
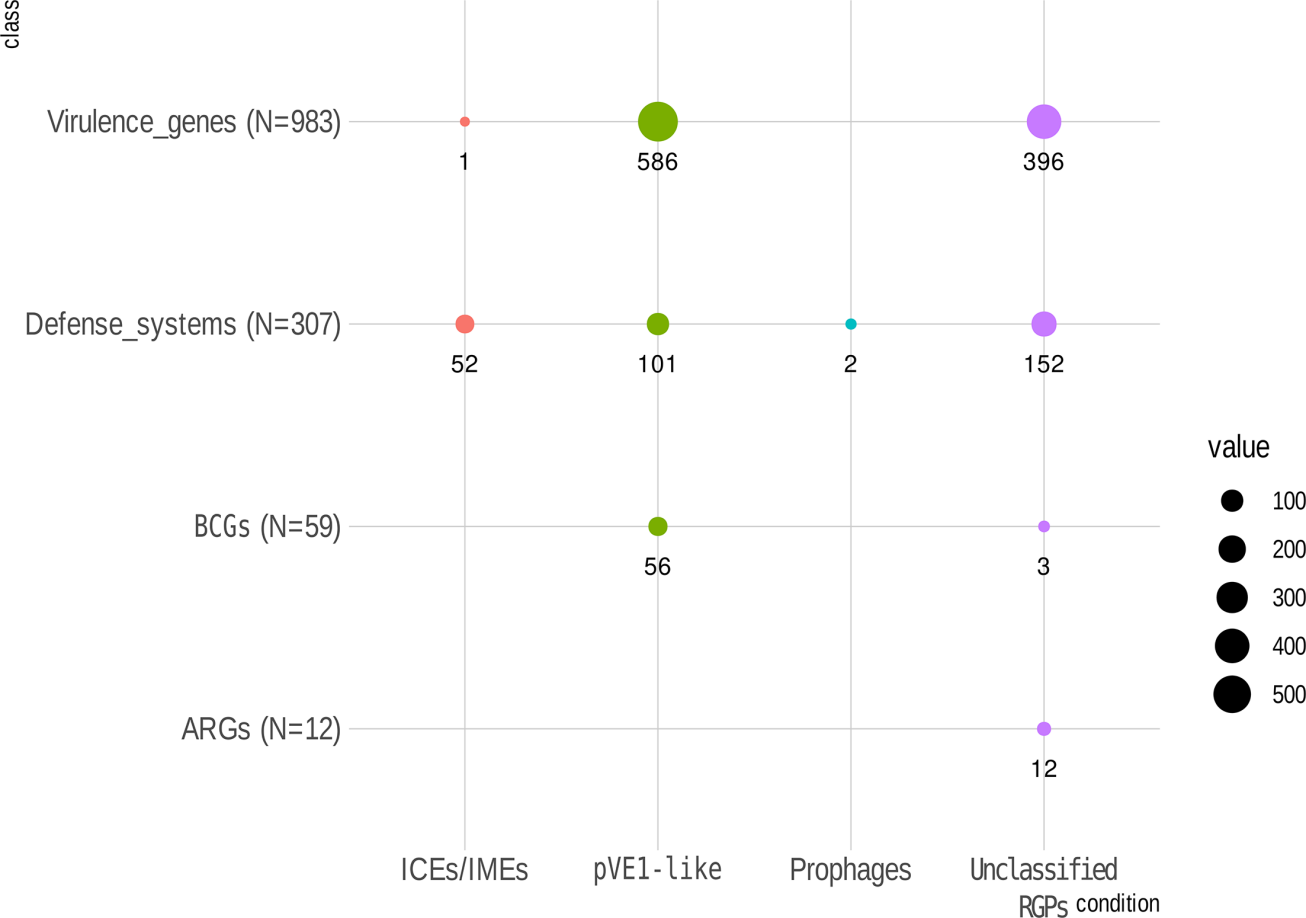
Number of genes related to virulence, anti-phage defence systems, BGCs and ARGs identified in the MGEs of the soft mobilome. pVE1 was the only plasmid encoding those genes. Dot’s size was proportional to the total number of genes identified.

Comparative analyses revealed that pVE1-like plasmids were the genetic elements of the accessory genome encoding the highest numbers of virulence genes, BGCs and a substantial number of anti-phage defence systems (101 of 307 systems identified) (Fig. 4). Interestingly, these genetic traits were not found in other *V. europaeus* plasmids outside the pVE1-like group. pVE1-like plasmids encoded 94.91% of the BGCs identified in the accessory genome. Regarding the BGC families (GCF) previously identified by Rodriguez *et al.* [9], pVE1-like plasmids harboured genes assigned to the GCF1/GCF2 and GCF9 families, which produce PKS-NRP hybrid polyketides and arylpolyene-NRPS hybrids, respectively (Fig. 3).

**Fig. 5.**
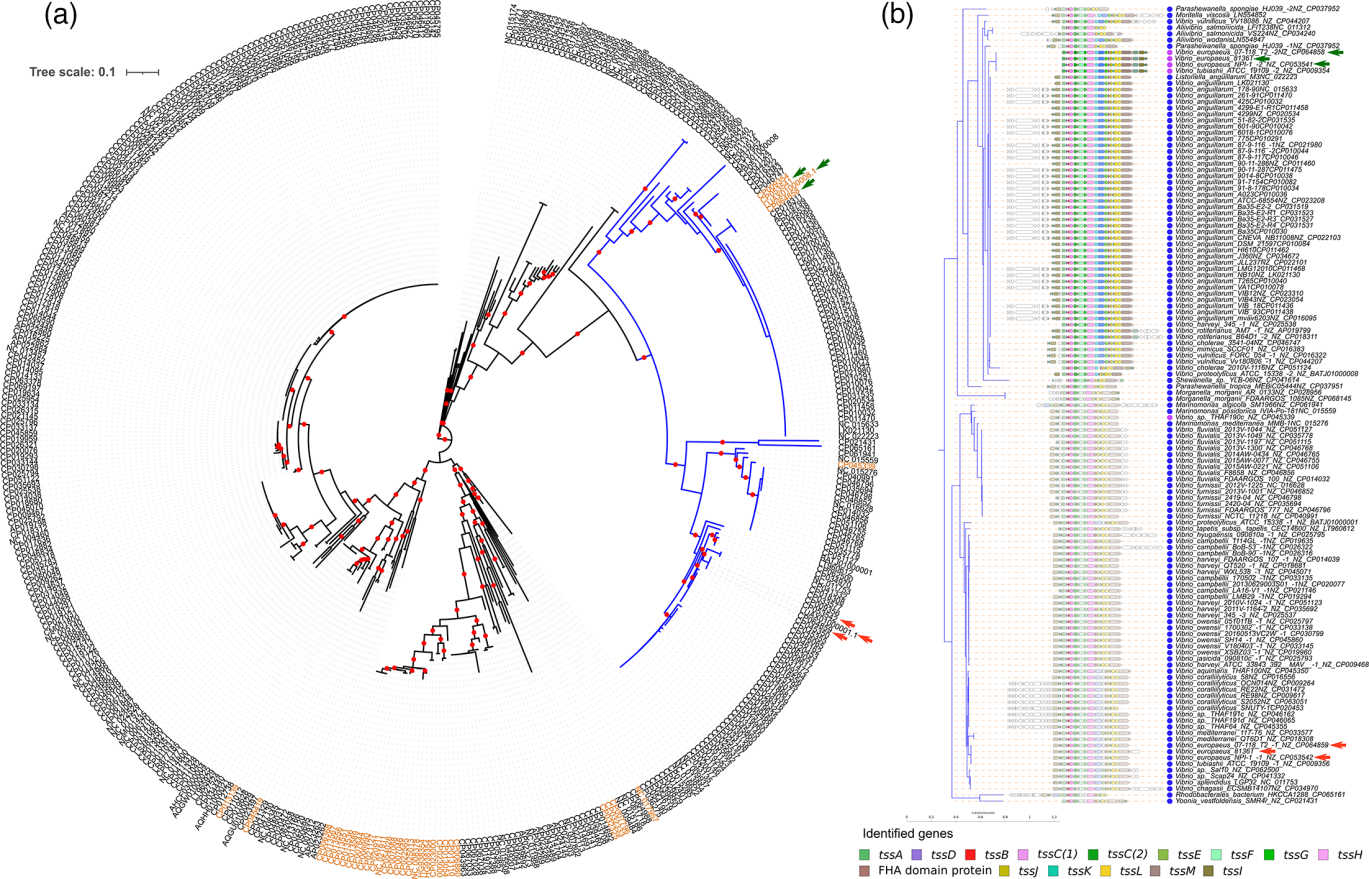
(**a**) Phylogenetic tree of T6SS i5 type based on the core TssB protein and (**b**) synteny and homology among T6SS core proteins (TssA-M). (**a**) Branches containing T6SS2 (green arrows) and T6SS1 (red arrows) were highlighted in blue. T6SSs encoded in a plasmid are depicted in orange. Red circles showed bootstrap values above 70. (**b**) Proteins with homology to the reference sequence (CECT8136) with an e-value cutoff of 1E^−15^ were colour-coded to represent different levels of homology. The intensity of the colours indicates the degree of homology with the reference sequence.

Furthermore, pVE1-like plasmids were the accessory genetic elements with the highest number of virulence genes, accounting for 59.61% of those associated with the accessory genome (Fig. 4). The most notable finding was the presence of a distinct T6SS, designated T6SS2, encoded in all pVE1-like plasmids and belonging to the T6SS i5 family (Figs3  5). *V. europaeus* also encodes two additional T6SSs on chromosome I (ChrI), T6SS1 and T6SS3, assigned to the core genome; notably, T6SS1 is also an i5 family member [9]. Consequently, both T6SS1 and T6SS2 were included in the phylogenetic analyses due to the relevance of their comparison (Fig. 5). Resultant phylogeny clustered both T6SSs in the same clade and revealed high synteny between T6SS1 and T6SS2, differing only in the position of the tube-spike genes (TssI/VgrG and PAAR) (Fig. 5b). Comparative analysis between core T6SS1 and T6SS2 proteins showed high amino acid sequence homology only for TssB1 vs. TssB2 (77.06%) and TssC1a vs. TssC2a (71.40%), whereas most other core proteins exhibited homology below 40% [e.g. TssA, TssC2(2), TssE, TssF, TssG, TssI/VgrG, TssJ, TssK, TssL and TssM]. This sequence divergence suggests that the two systems are functionally distinct, further supported by their placement on separate branches of the phylogenetic tree (Fig. 4b). Although other strains analysed also harboured two i5 family T6SSs, only *V. europaeus* and the closely related *V. tubiashii* ATCC 19109 carried both plasmidic and chromosomal versions of a T6SS i5 (Fig. 5a). pVE1-like plasmids also carry additional virulence genes, including the rtx cluster (*rtxB*, *rtxD* and *rtxE*) encoded by pVE1 of NPI-1, and single copies of the *cysC1* gene, identified in 13 plasmids (Fig. 3). Lastly, pVE1-like plasmids encode a substantial repertoire of anti-phage defence systems (32.90%) within the *V. europaeus* accessory genome (Figs3  4). Notably, Spanish and French isolates exhibited intrapopulation variation in defence profiles (e.g. sub-clusters Ia and Ib for Spanish strains; sub-clusters IIa–c and clusters III–VII for French strains), highlighting the high variability of phage defence systems even among closely related strains (Table S1, available in the online Supplementary Material, and Fig. 3). Among restriction-modification (RM) systems, type I RM systems were detected in strains 07/108 T1, CECT8426 and 07/120 T1. In contrast, toxin-antitoxin (TA) systems were rare, with only an AbiE/SanTA system identified in the pVE1 plasmid of strain EX1.

### Most chromosomic RGPs have identifiable insertion sites and were not assigned to known MGEs

**Fig. 6.**
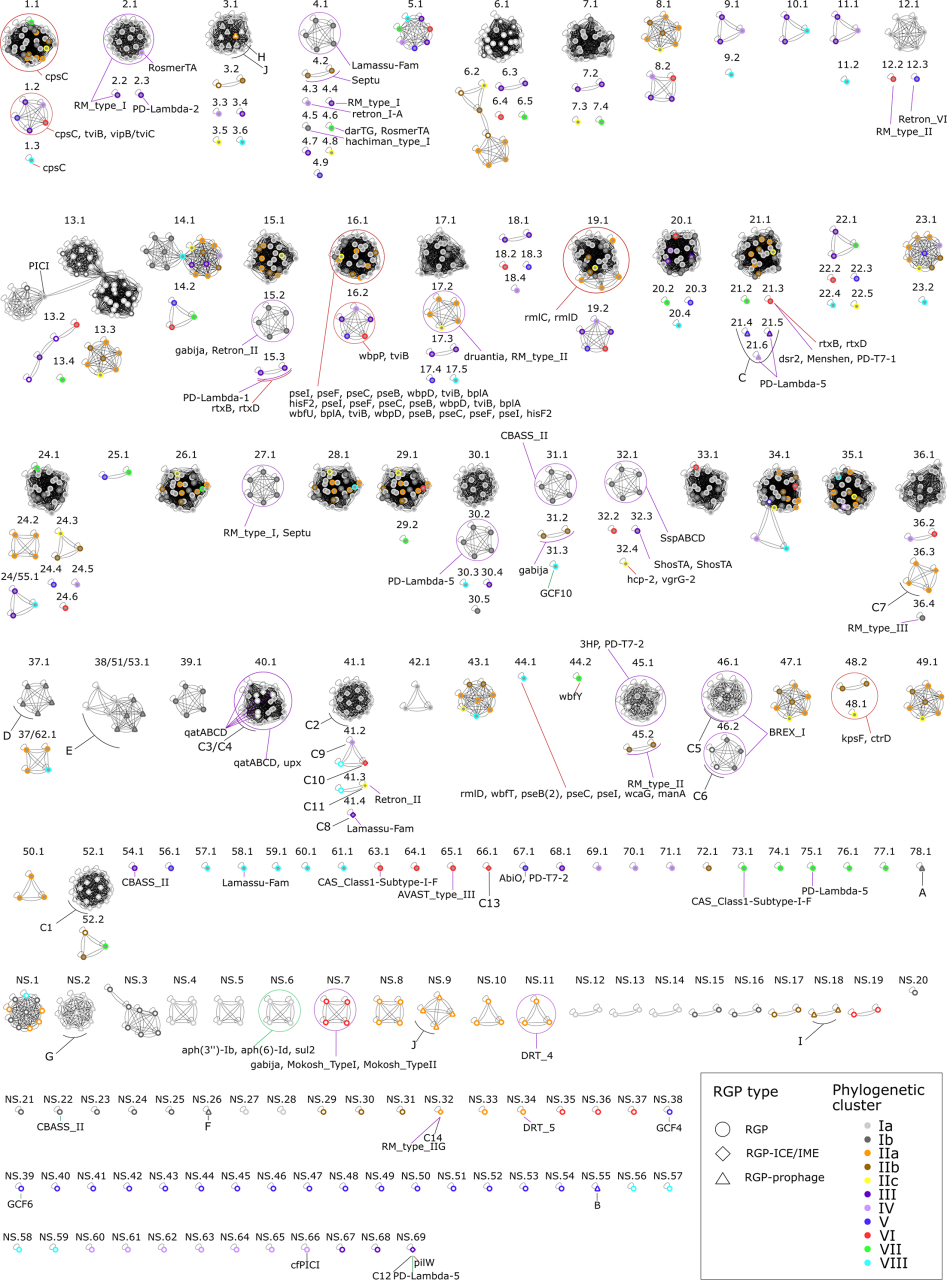
Network showing the 228 RGP families, 169 for contextualized chromosomic RGPs (named *x*.*y*, where *x* is the spot of insertion, e.g. 1–78, and *y* is the number to define a specific RGP family) and 69 for the decontextualized RGPs (NS.1–NS.69; 69 RGP families). Jaccard Index cutoff of 0.394 was used. Grey-filled nodes showed contextualized RGPs and white-filled nodes represented decontextualized RGPs. The colour of each node border identifies its phylogenetic cluster origin. Chromosomic RGPs assigned to an ICE/IME or a prophage are represented as rhombuses or triangles, respectively.

A total of 1,135 chromosomic RGPs were identified across *V. europaeus* genomes. Those showed a wide size range: >3–10 kb (*n*=531), 10–19 kb (*n*=335), 19–35 kb (*n*=180), 36–50 kb (*n*=81) and 50–66 kb (*n*=8). Classification of chromosomic RGPs was based on two criteria (Table 3): (i) whether a chromosomal insertion site was identified (contextualized RGPs) or not (decontextualized RGPs); and (ii) whether the RGP corresponded to or formed part of a known MGE (ICEs/IMEs, prophages or phage satellites) or not (unclassified chromosomal RGPs). Subsequently, a network of 228 RGP families was constructed (Fig. 6).

**Table 3.** Classification of the 1,135 chromosomal RGPs according to whether their insertion site was identified (contextualized RGPs) or not (decontextualized RGPs), or if they were found in MGEs or not (unclassified chromosomal RGPs) RGP families indicated the number of RGPs assigned to each family, for instance, 240 decontextualized RGPs were assigned to 69 RGP families (NS.1–NS.69).

		Classification	No.	RGP families	Additional information
**Have the spot of insertion been identified in the chromosome?**	No	*Decontextualized RGPs*	240	69	
	Yes	*Contextualized RGPs*	895	159	78 insertion spots: 53 spots containing multiple RGPs (3–39 RGPs) and 25 spots containing only one RGP.
**Are the chromosomic RGPs any known MGE?**	No	*Unclassified RGPs*	1,000		–
	Yes	*ICEs/IMEs*	78		–
		*Prophages*	55		–
		*Phage satellites*	2		–

According to the classification based on the spot of insertion, 895 out of the 1,135 chromosomic RGPs were identified as contextualized RGPs (Table 3). These 895 RGPs were distributed across 78 insertion sites. In detail, 53 sites contained multiple RGPs (3–39 RGPs), while 25 sites contained only one (Fig. 6 and Table 3). These contextualized RGPs were assigned to 159 families (numbered 1–78 in Fig. 6), with some families found at multiple sites, highlighting the dynamic role of insertion sites in shaping the accessory genome (24/55.1, 37/62.1 and 28/51/53.1; Fig. 6). In contrast, 240 RGPs (Table 3) were designated as decontextualized RGPs because no insertion site could be identified; these were classified into 69 RGP families (indicated as NS.1–NS.69 in Fig. 6). Decontextualized RGPs were predominantly identified from short-read assemblies, a finding supported by a positive correlation between genome fragmentation and the number of decontextualized RGPs (Fig. S1).

Considering an alternative classification criterion, assignment of chromosomic RGPs to known MGEs revealed that 78 were ICEs/IMEs, 55 were integrated into prophages and 2 were associated with phage satellites (Table 3). Strikingly, 1,000 chromosomal RGPs could not be assigned to any known MGEs and were therefore designated as unclassified (Table 3).

### Unclassified chromosomic RGPs are the main genetic reservoirs of anti-phage defence systems and ARGs

Examining RGPs not associated with any MGE (i.e. unclassified RGPs), these regions harboured a wide array of accessory genome traits, including a small number of secondary metabolite genes (5.1%), a significant proportion of virulence genes (40.3%), most defence systems (49.5%) and all antibiotic resistance genes (100%) (Fig. 4).

Regarding the first type, the three BGC singletons detected in RGPs (Fig. 6) were as follows: GCF10, related to butyrolactone production in strain 07/108 T1 (RGP family 31.3); and GCF4, encoding RiPPs, and GCF6, producing an NRPS, both found in the decontextualized families NS39 and NS38, respectively, in strain 071316 f. For virulence-related genes, a total of 396 were encoded by unclassified RGPs (Fig. 6). Their functions were primarily associated with immune evasion, including antiphagocytosis [capsular polysaccharide: *wbfU*, *cpsC*, *rmlC*, *rmlD*, *wbfY*, *wbfT*, *pseC*, *tviB*(2), *vipB/tviC*], adherence [lipopolysaccharide (LPS) O-antigen; type IV pili: *tviB*, *hisF2*] and glycosylation (system gene *pseB*). Others were linked to host-damaging factors such as toxins (RTX toxins: *rtxB*, *rtxD*) and endotoxins (LPS, LOS: *bplA*, *kpsF*). These virulence genes were distributed across 13 RGP families (1.1, 1.2, 1.3, 15.3, 16.1, 16.2, 19.1, 21.3, 32.4, 44.1, 44.2, 48.1 and 48.2) spanning 8 different insertion sites (Fig. 6).

As the most significant feature of unclassified RGPs, they carry the majority of traits associated with defence against phage predation and antimicrobial agents (Fig. 4). This group contained a total of 152 anti-phage defence systems (Fig. 4). Notably, nine insertion sites were identified as specific integration points for unclassified RGPs carrying anti-phage defence functions (Table 4 and Fig. 6). These insertion sites are defined as hotspots, as they serve as integration points for different RGP families encoding distinct anti-phage defence systems (Table 4 and Fig. 6). In contrast, the same insertion sites can harbour anti-phage defence genes together with genes of other functions within the same RGP, such as families 17.2 (*druantia* and *RM_type_II*), 27.1 (*RM_type_I* and *Septu*), 30.2 (*PD-Lambda-5*), 36.4 (*RM_type_III*), 40.1 (*qatABCD* and *upx*), 54.1 (*CBASS_II*), 58.1 (*Lamassu-Fam*), 63.1 (*CAS_Class1-Subtype-I-F*), 65.1 (*AVAST_type_III*), 67.1 (*AbiO* and *PD-T7-2*), 73.1 (*CAS_Class1-Subtype-I-F*) and 75.1 (*PD-Lambda-5*) (Fig. 6). Additionally, several decontextualized RGP families also carried anti-phage defence systems: NS.7 (*Gabija*, *Mokosh_TypeI* and *Mokosh_TypeII*), NS.11 (*DRT_4*), NS.22 (*CBASS_II*) and NS.34 (*DRT_5*) (Fig. 6). Unclassified RGPs were the only regions of plasticity encoding antibiotic resistance genes (Fig. 4). The RGP family NS.6 was the only one associated with this function and harboured the genes *aph(3″)-Ib*, *aph(6)-Id* and *sul2*, which confer resistance to streptomycin and sulphonamide, and were detected in strains 3454, 3492, 3610 and 3614 (Fig. 6). This finding highlights the possible role of specific mobile genomic regions in driving antibiotic resistance in *V. europaeus*.

**Table 4.** Hotspots for the exclusive insertion of unclassified RGPs carrying anti-phage defence functions Anti-phage defence systems associated with an RGP family are shown in brackets.

Spot of insertion	RGP family and anti-phage defence system identified
Spot 2	2.1 (RosmerTA), 2.2 (RM_type_I) and 2.3 (PD-Lambda-2)
Spot 4	4.1 (Lamassu-Fam), 4.2 (Septu), 4.3 (retron_I-A), 4.4 (RM_type_I), 4.5 (Hachiman_type_I) and 4.6 (darTG and RosmerTA)
Spot 12	12.2 (RM_type_II) and 12.3 (Retron_VI)
Spot 15	15.2 (Gabija and retron II) and 15.3 (PD-Lambda-1)
Spot 31	31.1 (CBASS_II) and 31.2 (Gabija)
Spot 32	32.1 (SspABCD) and 32.3 (ShosTA)
Spot 41	41.1 and 41.2 (both with BREX_I)
Spot 45	45.1 (3HP and PD-T7-2) and 45.2 (RM_type_II)
Spot 46	46.1 and 46.2 (both with BREX_I)

### The primary role of ICEs/IMEs is related to anti-phage defence functions

Focusing on the group of RGPs classified as ICEs/IMEs, a total of 78 chromosomal RGPs were identified within this category of MGEs, ranging in size from 7.86 to 57.45 kb. Classification as ICEs/IMEs was based on the positive detection of both integrases and relaxases in their sequences. These regions of plasticity were widespread among *V. europaeus* strains, being detected in 25 of the 39 genomes examined (Table S2).

**Fig. 7.**
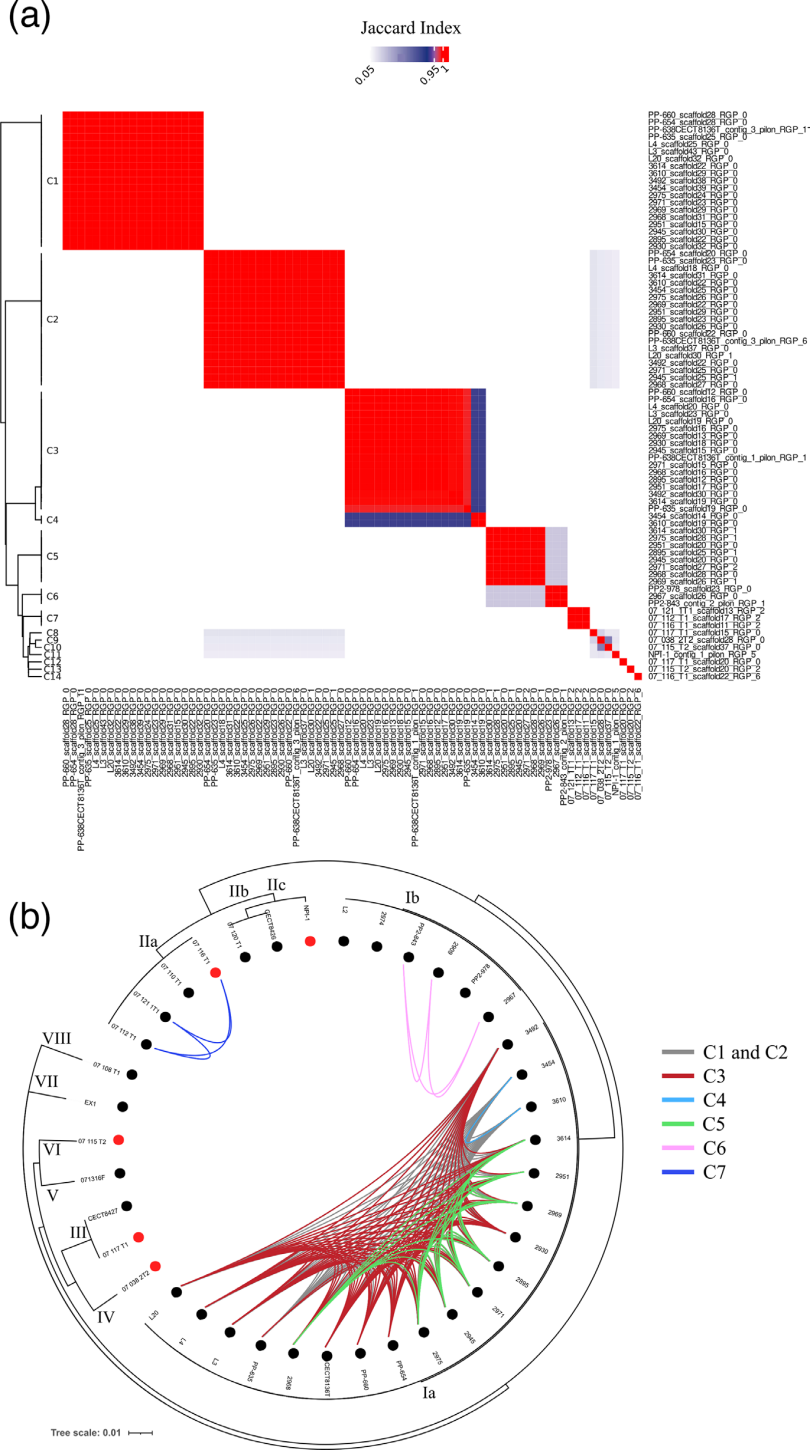
(**a**) Heatmap showing the results of the pairwise comparisons among ICEs/IMEs (clusters C1–C14) based on the Jaccard Index. (**b**) Distribution of ICEs/IMEs across *V. europaeus* strains. Clusters (C1–14) were defined using an index cutoff of 0.95. The strains were ordered according to the phylogeny based on the core genome [9], indicated on the outer ring. The red dot indicated the presence of unique ICEs/IMEs in a certain strain.

Clustering the ICEs/IMEs based on their similarity resulted in 14 clusters (C1–C14). These clusters were not ubiquitous and were instead associated with specific phylogenetic groups or with single strains (Fig. 7a): (i) clusters C1 and C2 were found in strains of phylogenetic sub-cluster Ia; (ii) clusters C3 and C4 were partially distributed within sub-cluster Ia, with C3 present in most strains of this group except for strains 3610 and 3614, which carried C4; (iii) cluster C5 was present in a subset of sub-cluster Ia strains (3614, 2975, 2971, 2969, 2968, 2951, 2945 and 2895); (iv) cluster C6 was detected in a subset of sub-cluster Ib strains (PP2-978, PP2-843 and 2967); (v) cluster C7 was identified in 75% of sub-cluster IIa strains (07_121_1T1, 07_116_T1 and 07_112_T1); and (vi) the remaining ICE/IME clusters corresponded to singletons: C8 and C12 in 07/117 T1; C9 in 07/038 2T2; C10 and C13 in 07/115 T2; C11 in NPI-1; and C14 in 07/116 T1 (Fig. 7a). The distribution of ICE/IME clusters suggests that strains within phylogenetic groups Ia, Ib and IIa are more closely related, as they consistently shared specific cluster types across members of each respective group.

Although 54 ICEs/IMEs matched elements deposited in the ICEberg3 database, the total similarity scores were low in most comparisons (Table S2). Only ICEs/IMEs assigned to clusters C5 and C6 obtained scores higher than 5 – specifically, 9.5 for C5 and 10 for C6 – both of which resembled ARG-carrying ICEs/IMEs (SXT), such as ICEVchBan9 from *Vibrio cholerae* MJ-1236 (Table S2). These results suggest limited similarity between most ICEs/IMEs in *V. europaeus* and known elements in the ICEberg3 database, indicating that genomic data from this species could substantially enrich such repositories. For most ICEs/IMEs, insertion sites were identified, with the exception of C12 (NS.69) and C14 (NS.32), which were not assigned to any site (Fig. 6). Interestingly, some sites acted as hotspots for the insertion of multiple ICEs/IMEs (Fig. 6): (i) site 41, which hosted C2 (RGP family 41.1), C8 (RGP family 41.4), C10 and C9 (RGP family 41.2) and C11 (RGP family 41.3); and (ii) site 40, which hosted C3 and C4 (RGP family 40.1). ICEs/IMEs assigned to C12, C13 and C14 were singletons (NS.69, 66.1 and NS.32, respectively) (Fig. 6). As previously observed for unclassified RGPs, certain sites appear to specialize in the exchange and mobility of this type of MGE.

Regarding the specific functions identified within this class of MGEs, ICEs/IMEs encoded only 0.1% of the virulence genes in the accessory genome of *V. europaeus* (Fig. 4). Only the ICE/IME NS.69 (cluster C12) carried a virulence gene, the *pilW* gene, which is associated with adherence and type IV pili. In contrast, the primary role of ICEs/IMEs was related to anti-phage defence, encoding 16.9% of the defence systems in the accessory genome (Fig. 4), only outclassed by the unclassified RGPs. ICEs/IMEs contained a variety of anti-phage defence systems, differing from those present in the unclassified RGPs: C3 and C4 (qatABCD and qatABCD and upx, respectively; RGP family 40.1), C11 (Retron II; RGP family 41.3), C8 (Lamassu-Fam; RGP family 41.4), C5 and C6 (BREX I; RGP family 46.1 and 46.2), C14 (RM_type_2G; RGP family NS.32) and C12 (PD-Lambda-5; RGP family NS.69) (Fig. 6).

### Prophages and phage satellites do not contribute to bacterial virulence, antibiotic resistance or secondary metabolite production

**Fig. 8.**
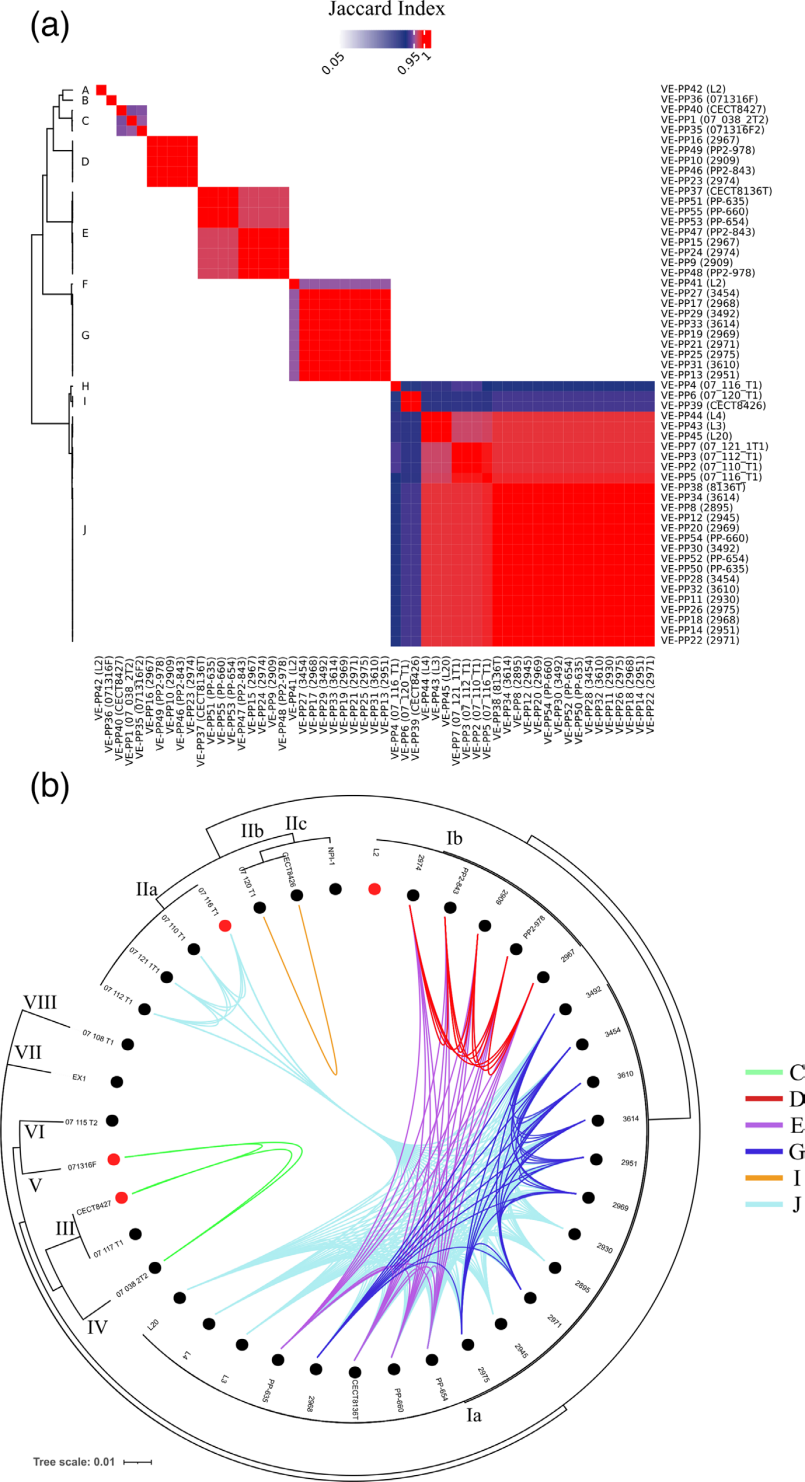
(**a**) Heatmap showing the results of pairwise comparisons among prophages. (**b**) Distribution of prophages among the *V. europaeus* genomes. Features were the same as in Fig. 7.

A total of 55 complete prophages could be identified from the 34 *V*. *europaeus* genomes analysed (Table S3). These prophages were clustered into ten groups (prophage clusters A–J) using a Jaccard similarity index cutoff of 0.95 (Fig. 8a). Some clusters were represented by only a single prophage, such as clusters A, B, F and H (Fig. 8a and Table S3). In contrast, the remaining clusters included at least two prophages: C (*n*=3), D (*n*=5), E (*n*=9), G (*n*=9), I (*n*=2) and J (*n*=23) (Fig. 8a and Table S3). Regarding taxonomic affiliation, prophages of clusters A and E showed similarity with *Vibrio* phage L9 and *Vibrio* phage 1.159.O._10 N.261.46.F12, whereas F and G with *Vibrio* phage ST2-2pr, and clusters H and J with *Inoviridae* sp. prophages (Table S3). The remaining prophages could not be associated with any known prophages and appear to be exclusive to *V. europaeus*, thereby expanding the current catalogue of identified prophages.

Prophage families showed a clear association with the phylogenetic clustering reported by Rodriguez *et al.* [9]. Examining specific genomic regions among the Spanish strains, only EX-1 (1985 Hatchery A) lacked any identified prophage in its genome (Table 1 and Fig. 8b). All the 19 Spanish strains from sub-cluster Ia were infected by prophages from cluster J (Fig. 8b), some presenting only prophages from this group, i.e. strains L20, L4, L3, 2930 and 2895, or in combination with other cluster prophages, such as (i) strains isolated in 2001 (PP-635, PP-660, PP-654 and CECT8136) carried prophages from clusters J and E; (ii) strains 3492, 3454, 3610, 3614, 2951, 2969, 2971, 2975 and 2968 harboured phages from clusters J and G (Fig. 8b). Prophages from cluster E were exclusive to Spanish strains but associated with both sub-clusters (Fig. 8 and Table 3, Supplementary Material 1): sub-cluster Ia (mentioned above) and sub-cluster Ib (2967, 2974, 2909, PP2-843 and PP2-978) (Fig. 8b). Regarding prophages that exclusively appeared in strains of sub-cluster Ib (Fig. 8b): (i) A and F prophages parasitized only the strain L2; and (ii) strains PP2-843, PP2-978, 2909, 2967 and 2974 were infected by the prophages from cluster D.

In the case of the French strains, no prophages were detected in strains 07/108 T1, 07/115 T2 and 07/117 T1 (Fig. 8b). Strain 07/116 T1 carried two prophages (clusters H and J), whereas the remaining French strains harboured only one. For example, cluster I prophages were found exclusively in French strains belonging to sub-cluster IIc (CECT8426 and 07/120 T1) (Fig. 8b). Only prophages from clusters C and J were detected in strains isolated from different countries (Fig. 8b): cluster C prophages infected French and American strains (phylogenetic clusters III, IV and V), while cluster J prophages were the only ones shared between Spanish and French strains (Fig. 8b). A detailed analysis of this prophage family revealed intra-specific variability within cluster J, supported by differences in genome size: prophages infecting French strains were ~6 kb, whereas those infecting Spanish strains were larger, around 14.6 kb. This observation was further supported by the Jaccard index (Table S3).

Surprisingly, no virulence factors, ARGs or BGCs were encoded by the prophages and only two prophages – VE-P40 and VE-P1 (cluster C) – encoded anti-phage defence systems (Fig. 4).

Of the 55 chromosomal RGPs identified as prophages (Table 3 and Fig. 6), insertion sites could be identified for some prophages (clusters A, C, D, E, H and J), whereas others were classified as decontextualized RGPs (clusters B, F, G, I and six prophages from cluster J) (Fig. 6 and Table S4). As observed for other genetic elements, the same insertion hotspot can harbour different RGP families. For example, site 21 was a hotspot for the three RGPs forming cluster C (21.4, 21.5 and 21.6), which shared the same region with anti-phage defence functions (PD-Lambda-5), and in addition, this spot could carry other RGPs with anti-phage functions and virulence factors not in prophages (21.3) (Fig. 6). By contrast, prophages from cluster E included RGPs belonging to the same family but inserted at different spots (38, 51 and 53.1) (Fig. 6), indicating that these systems are widely distributed and frequently co-located with other genetic elements throughout the genome. Finally, only two phage satellites were identified (Fig. 6): (i) a cfPICI family microsatellite in strain 07/038 2T2 (RGP family NS.66); and (ii) a PICI family microsatellite in strain PP-635 (RGP family 13.1). As expected, these RGPs were not associated with any known functional categories.

### The soft mobilome represents most of the accessory genome and a significant portion of the *V. europaeus* pangenome

A dual approach was used to establish the mobilome of *V. europaeus*.

**Fig. 9.**
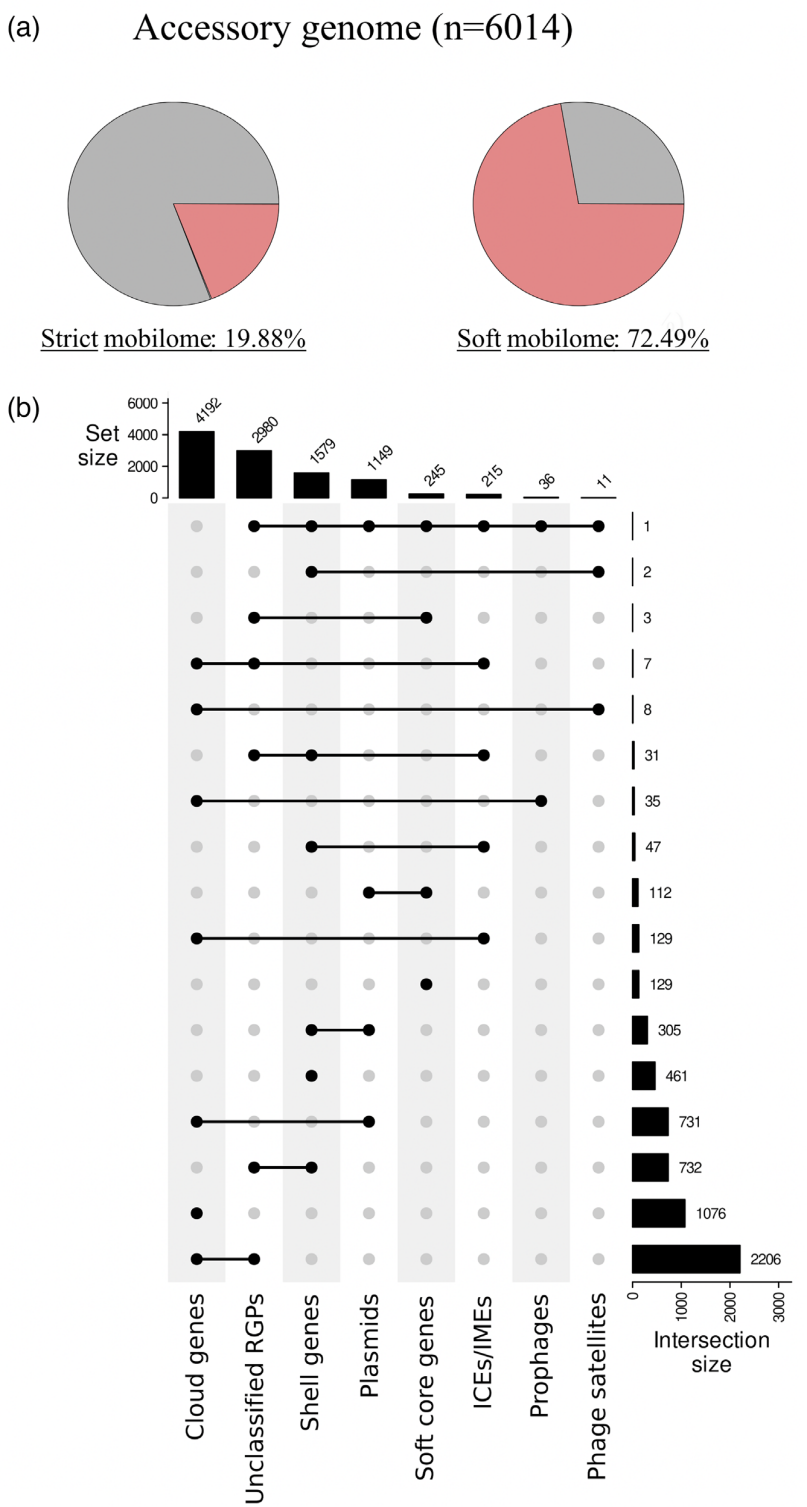
Structural organization of accessory genome of *V. europaeus*. (**a**) Percentage of the accessory genome assigned to strict and soft mobilome. (**b**) Scatter plot showing the accessory fractions (cloud genes, shell genes and soft-core genes) associated with plasmids, prophages, phage satellites and unclassified chromosomic RGPs.

1. A strict mobilome, comprising 19.88% of the accessory genes (1,196/6,014 genes) assigned to well-characterized MGEs identified in this study, such as mobile plasmids, ICEs/IMEs, prophages and phage satellites. Strict mobilome constituted 12.13% (1,196/9,860 genes) of the total pangenome (Fig. 9a).
2. A soft mobilome, a much more flexible concept including unclassified chromosomic RGPs as unidentified MGEs. Unclassified chromosomic RGPs accounted for 63% of the accessory genes (3,789/6,014 genes) and, notably, 52.39% (2,206/4,210 genes) of the cloud genes. This is important since the cloud fraction of the accessory genome is usually acquired from HGT events [10]. The cloud genome was indeed the predominant fraction among MGEs of the strict mobilome, encompassing most of the genes associated with prophages (97%), plasmids (64%), ICEs/IMEs (60%) and phage satellites (73%) (Fig. 9b). All these reasons supported the inclusion of unclassified chromosomic RGPs in the soft mobilome, even though no known mobility genes (e.g. relaxases, integrases/recombinases) were detected in unclassified chromosomic RGPs. Under this last assumption, the proportion of accessory genes assigned to the soft mobilome increased to 73.01% (4,391/6,014 genes), representing 44.53% (4,391/9,860 genes) of the *V. europaeus* pangenome (Fig. 9a).

## Discussion

Bacterial sampling in veterinary and aquaculture settings is often biased towards moribund or dead animals, as these are typically targeted for pathogen isolation. In our dataset, only strain 071316F was obtained from a natural environment – seawater in Netarts Bay, near a hatchery effluent [48] – whereas the remaining *V. europaeus* strains originated from artificial environments such as shellfish hatcheries and mortality outbreaks. This sampling bias likely reflects the limited availability of environmental isolates. Nevertheless, our dataset encompasses strains from diverse geographical origins, hosts and years of isolation, providing a representative overview of *V. europaeus* genomic diversity.

This study provides, indeed, the first comprehensive characterization of the accessory genome in a shellfish aquaculture pathogen belonging to the *Vibrio* genus. Within the accessory genome, unclassified chromosomal RGPs emerged as one of the principal genetic elements shaping the species’ evolutionary dynamics in *V. europaeus*. Those genetic elements were included in the soft mobilome as potential or ancient MGEs because (i) RGPs may progressively lose their capacity to mobilize due to inactivation by random mutations or deletions of the genes responsible for mobility mechanisms [29]; (ii) RGPs can be mobilized through mechanisms that remain unidentified [49]; (iii) RGPs may not be detected as mobile elements due to the loss of mobility genes located at the edges of contigs in incomplete assemblies [10]; and (iv) RGPs account for the majority of cloud genes and contribute substantially to the observed genomic variability.

The functional characterization of the accessory genome revealed an association of virulence genes, anti-phage defence systems, secondary metabolite biosynthetic genes and antibiotic resistance genes with MGEs included in the soft mobilome (e.g. ICEs/IMEs, plasmids, prophages, phage satellites and most unclassified chromosomic RGPs). The mobilization of these genes via MGEs may promote rapid shifts in phenotypic traits, suggesting a rapid turnover of such genes within *V. europaeus* populations, independent of the more conserved physiological and metabolic functions encoded by the core genome [50].

Focusing on the different functions described, 73% of the anti-phage defence systems were located on MGEs belonging to the soft mobilome. This percentage closely matches the 72% reported in *Vibrio crassostreae*, supporting the evidence that HGT events play a major role in the emergence of phage resistance through the acquisition of MGEs [51]. Hussain *et al.* [50] demonstrated that phage resistance is one of the most important selective forces shaping clonal diversity. Similarly, Rodriguez *et al.* [9] reported the existence of clonal strains with different phage defence systems within our *V. europaeus* dataset, particularly those strains belonging to phylogenetic cluster Ia isolated from Hatchery B (Table S5). As also observed by Hussain *et al.* [50], this variability of phage defence systems can explain a substantial portion of the accessory genome, suggesting that phages play a significant role in shaping clonal diversity also in *V. europaeus*. Chromosomic MGEs – unclassified RGPs, ICEs/IMEs and prophages – were enriched in insertion hotspots with higher activity than the rest of the genome in terms of acquiring new elements, showing a strong tendency to easily acquire RGP families encoding anti-phage defence systems. This provides a highly diverse repertoire of defence genes that support the survival of *V. europaeus*, enabling rapid turnover of these systems. This phenomenon has been observed in other species, such as *Escherichia coli* and *Pseudomonas aeruginosa*, but has not previously been described in marine vibrios [49,52]. The clustering of defence systems within these genetic elements is likely shaped by selective pressures that favour host fitness in phage-rich environments, such as marine ecosystems, for instance, aquaculture facilities.

Unclassified RGPs were the only genetic elements encoding antimicrobial resistances (AMRs), suggesting their potential role in bacterial survival and environmental adaptation to these compounds. A second function associated with chromosomic unclassified RGPs was related to bacterial virulence, encoding 40% of the virulence genes associated with the accessory genome. The virulence genes encoded by unclassified chromosomal RGPs are primarily involved in polysaccharide synthesis (insertion hotspot 16). These polysaccharides play crucial roles in forming cell surface structures such as the O-chain of LPSs and capsular polysaccharides, as well as in glycosylating external features like flagella.

pVE1-like plasmids were only found in *V. europaeus* strains and its closest relative *V. tubiashii* (p251), highlighting for the first time the significance of this MGE in the biology of both pathogens. pVE1-like plasmids are the largest and most ubiquitous MGEs in *V. europaeus*, with only strain 07/115 T2 lacking this plasmid. Although it is generally accepted that carrying a plasmid incurs a fitness cost [18,21, 53, 54], the widespread maintenance of pVE1-like plasmids in the studied population suggests that their numerous features – compared with other MGEs – such as virulence factors, anti-phage defence mechanisms and BGCs, outweigh the associated costs under the ecological conditions encountered by *V. europaeus*. These genes are strongly linked to adaptation to novel environments and conditions, providing a selective advantage for *V. europaeus* [18]. The presence of different anti-phage defence systems encoded within the same pVE1 plasmid could be explained by (i) prevention of the entry of foreign DNA, such as new phages and other mobile elements [55], or (ii) plasmid stabilization during bacterial division through post-segregation killing or addiction mechanisms [56]. However, the identification of addiction systems potentially involved in plasmid stability was very limited. For instance, RM systems, such as type I RM systems [57,59], were detected in strains 07/108 T1, CECT8426 and 07/120 T1, while a TA system, such as AbiE/SanTA [56,60], was identified only in the pVE1 plasmid of strain EX1. Among virulence factors, all pVE1 plasmids encoded their own T6SS, designated as T6SS2 and closely related to the T6SS i5 found in *V. crassostreae* J5-20 genome, also encoded in a plasmid (pGV) [61]. In *V. crassostreae*, pathogenicity is linked to a novel transcriptional regulator that activates the bidirectional promoter of a T6SS gene cluster, triggering cytotoxicity in oyster haemocytes [61]. Remarkably, *V. europaeus* harbours two core T6SSs (T6SS1 and T6SS3) on chromosome I in addition to T6SS2. The distribution of T6SSs in the genome of *V. europaeus* is noteworthy, given the general absence of chromosomal T6SSs in bacteria carrying T6SS-encoding plasmids [62]. Finally, pVE1-like plasmids encoded the majority of BGCs associated with RGPs, supporting their importance for the adaptation of *V. europaeus*. For instance, the arylpolyene-NRPS hybrid cluster (GCF9) is likely associated with colonization through chitin utilization [63]. In relation to the plasmid mobility, although most pVE1-like plasmids were conjugative, others, such as the pVE1 from strains NPI-1 and 07/108 T1, were mobilizable due to the absence of MPF and T4CP [26]. This transition from conjugative to mobilizable often involved gene loss and a reduction in plasmid size, which could be associated with the presence of transposable elements within the plasmid [64]. This hypothesis is supported since pVE1-like plasmid harboured by NPI-1 was the smallest plasmid within its phylogenetic cluster, and pVE1 in the strain 07/108 T1 also lacked BGCs.

Prophages and phage satellites did not carry genes related to virulence, secondary metabolite production or AMR. Only a small fraction (0.6%) of anti-phage defence systems were encoded by prophages. Prophages integrated into the genome are usually vulnerable to predation of their bacterial host by exogenous phages; in response, these MGEs could encode different anti-phage systems to protect host cells without impairing their ability to replicate lytically [19]. Among anti-phage defence systems identified in this study, the PD-Lambda-1, PD-Lambda-2, PD-Lambda-5, PD-T7_1, PD-T7_2 and DarTG systems are typical of prophages [19]. However, only two out of eight PD-Lambda-5 systems were encoded into prophages, supporting that detection of prophages could be underestimated due to the strict parameters established for the identification of prophages. Moreover, the presence of PD-T7_1 and PD-Lambda-1 in some pVE1 plasmids suggested possible integration of prophages into their flexible defence islands [19,20].

Although the genome dataset used is focused towards moribund or dead animals from hatchery environments, the evolutionary dynamics and ecological adaptability of *V. europaeus* appear to be strongly influenced by its accessory genome, with most of the accessory genes encoded by MGEs within a soft mobilome concept. The functional characterization of these MGEs revealed that they act as major reservoirs of virulence genes, anti-phage defence systems, secondary metabolite biosynthetic genes and antibiotic resistance genes in aquaculture settings. Together, these findings suggest that the accessory genome plays a pivotal role in the rapid genetic turnover and adaptive potential of *V. europaeus* populations in aquaculture environments.

## Supplementary material

10.1099/mgen.0.001600 Supplementary Material 1.

## Abbreviations

AMR: antimicrobial resistance
ARGs: antimicrobial resistance genes
BGCs: biosynthetic gene clusters
HGT: horizontal gene transfer
ICEs/IMEs: integrative and conjugative/mobilizable elements
LPS: lipopolysaccharide
MGEs: mobile genetic elements
MPF: mating pair formation
PICIs: phage-inducible chromosomal islands
PTU: plasmid taxonomic unit
RGPs: regions of genomic plasticity
RM: restriction-modification
TA: toxin-antitoxin
