# Supplementary material for: Genomic plasticity and mobilome architecture of Vibrio europaeus reveal key mechanisms of evolutionary adaptation
Source: Microb Genom. 2026 Jan 21;12(1):001600. doi: 10.1099/mgen.0.001600 (PMC12824646; doi:10.1099/mgen.0.001600)
Supplement: Supplementary Material 1. [file mgen-12-01600-s001.pdf]

Accessory genetic elements drive the adaptation and evolution of the shellfish pathogen *Vibrio europaeus* as reservoir of virulence genes, anti-phage defense systems, and secondary metabolites.

Sergio Rodriguez, Diego Rey-Varela, Clara Martinez, Paulino Martínez, Marie-Agnés Travers, Juan L. Barja, Javier Dubert

Supplementary Material

Table S1. Profiles of anti-phage defense systems encoded by the strains belonging to a certain phylogenetic cluster.
Exceptions are indicated in brackets. Cluster VI was not included because it was constituted by the unique strain without pVE1-like plasmids (07/115 T1).

**Phylogenetic cluster based on the core-genome:**	**Defense profiles:**
subcluster Ia	Gao_Hhe system, Gabija and PD-T7-1 systems
subcluster Ib	Gao_Hhe system, Mokosh_TypeII systems and PD-T4-7 system (strains 2909 and 2967 lacked PD-T4-7 system)
Subcluster IIa	Gao_Hhe system, DRT_4 systems, PD-Lambda-1, and Mokosh_TypeII (strain 07/116 T1 lacked both copies of DRT_4)
Subcluster IIb	Gao_Hhe system, CBASS_I, Kiwa, and RM_Type_I systems
Subcluster IIc	Gao_Hhe system, Lamassu-Fam and Mokosh_Type II systems
Cluster III	Gao_Hhe system, Lamassu-Fam, PD-T7-1, and Mokosh_TypeII systems (strain 07_117_T1 lacked PD-T7-1)
Cluster IV	Gao_Hhe system, Mokosh_TypeII system
Cluster V	Gao_Hhe system, NLR_like_bNACHT09 and Mokosh_TypeII systems
Cluster VI	Gao_Hhe system, Hachiman and SanaTA systems
Cluster VII	Gao_Hhe system, RM_Type_I and Dnd_ABCDEFGH systems

Table S2. List of ICEs/IMEs identified from the *V. europaeus* genomes.
ICE/IME clusters (C1-C14), the integrase and the relaxase were showed on Table. Similarities against ICEberg3 (ICEberg IDs were showed in parentheses) were established in bases of the total score (TS) and Cumulative Blast bit score (CBBS).

Cluster	RGP	Lenght (kb)	Integrase	Relaxase	Match	TS	CBBS
C1	PP-660_scaffold28_RGP_0	8.15	Phage_integrase	T4SS_MOBV	-	-	-
C1	PP-654_scaffold28_RGP_0	8.15	Phage_integrase	T4SS_MOBV	-	-	-
C1	CECT8136T_contig_3_RGP_11	8.15	Phage_integrase	T4SS_MOBV	-	-	-
C1	PP-635_scaffold25_RGP_0	8.15	Phage_integrase	T4SS_MOBV	-	-	-
C1	L4_scaffold25_RGP_0	8.15	Phage_integrase	T4SS_MOBV	-	-	-
C1	L3_scaffold43_RGP_0	8.15	Phage_integrase	T4SS_MOBV	-	-	-
C1	L20_scaffold32_RGP_0	8.15	Phage_integrase	T4SS_MOBV	-	-	-
C1	3614_scaffold22_RGP_0	8.15	Phage_integrase	T4SS_MOBV	-	-	-
C1	3610_scaffold29_RGP_0	8.15	Phage_integrase	T4SS_MOBV	-	-	-
C1	3492_scaffold38_RGP_0	8.15	Phage_integrase	T4SS_MOBV	-	-	-
C1	3454_scaffold39_RGP_0	8.15	Phage_integrase	T4SS_MOBV	-	-	-
C1	2975_scaffold24_RGP_0	8.15	Phage_integrase	T4SS_MOBV	-	-	-
C1	2971_scaffold23_RGP_0	8.15	Phage_integrase	T4SS_MOBV	-	-	-
C1	2969_scaffold29_RGP_0	8.15	Phage_integrase	T4SS_MOBV	-	-	-
C1	2968_scaffold31_RGP_0	8.15	Phage_integrase	T4SS_MOBV	-	-	-
C1	2951_scaffold15_RGP_0	8.15	Phage_integrase	T4SS_MOBV	-	-	-
C1	2945_scaffold30_RGP_0	8.15	Phage_integrase	T4SS_MOBV	-	-	-
C1	2930_scaffold32_RGP_0	8.15	Phage_integrase	T4SS_MOBV	-	-	-
C1	2895_scaffold22_RGP_0	8.15	Phage_integrase	T4SS_MOBV	-	-	-
C2	PP-660_scaffold22_RGP_0	15.13	Phage_integrase	profile_MOBM	Trb-1 (1091)	1	263
C2	PP-654_scaffold20_RGP_0	15.13	Phage_integrase	profile_MOBM	Trb-1 (1091)	1	263
C2	CECT8136T_contig_3_RGP_6	15.13	Phage_integrase	profile_MOBM	Trb-1 (1091)	1	263
C2	PP-635_scaffold23_RGP_0	15.13	Phage_integrase	profile_MOBM	Trb-1 (1091)	1	263
C2	L4_scaffold18_RGP_0	15.13	Phage_integrase	profile_MOBM	Trb-1 (1091)	1	263
C2	L3_scaffold37_RGP_0	15.13	Phage_integrase	profile_MOBM	Trb-1 (1091)	1	263
C2	L20_scaffold30_RGP_1	15.13	Phage_integrase	profile_MOBM	Trb-1 (1091)	1	263
C2	3614_scaffold31_RGP_0	15.13	Phage_integrase	profile_MOBM	Trb-1 (1091)	1	263
C2	3610_scaffold22_RGP_0	15.13	Phage_integrase	profile_MOBM	Trb-1 (1091)	1	263
C2	3492_scaffold22_RGP_0	15.13	Phage_integrase	profile_MOBM	Trb-1 (1091)	1	263
C2	3454_scaffold25_RGP_0	15.13	Phage_integrase	profile_MOBM	Trb-1 (1091)	1	263
C2	2975_scaffold26_RGP_0	15.13	Phage_integrase	profile_MOBM	Trb-1 (1091)	1	263
C2	2971_scaffold25_RGP_0	15.13	Phage_integrase	profile_MOBM	Trb-1 (1091)	1	263

C2	2969_scaffold22_RGP_0	15.13	Phage_integrase	profile_MOBM	Trb-1 (1091)	1	263
C2	2968_scaffold27_RGP_0	15.13	Phage_integrase	profile_MOBM	Trb-1 (1091)	1	263
C2	2951_scaffold29_RGP_0	15.13	Phage_integrase	profile_MOBM	Trb-1 (1091)	1	263
C2	2945_scaffold25_RGP_1	15.13	Phage_integrase	profile_MOBM	Trb-1 (1091)	1	263
C2	2930_scaffold26_RGP_0	15.13	Phage_integrase	profile_MOBM	Trb-1 (1091)	1	263
C2	2895_scaffold23_RGP_0	15.13	Phage_integrase	profile_MOBM	Trb-1 (1091)	1	263
C3	PP-660_scaffold12_RGP_0	47.42	Phage_integrase	T4SS_MOBP1	CMGI-3 (338)	4.5	953
C3	PP-654_scaffold16_RGP_0	47.42	Phage_integrase	T4SS_MOBP1	CMGI-3 (338)	4.5	953
C3	CECT8136T_contig_1_RGP_1	47.42	Phage_integrase	T4SS_MOBP1	CMGI-3 (338)	4.5	953
C3	PP-635_scaffold19_RGP_0	47.42	Phage_integrase	T4SS_MOBP1	CMGI-3 (338)	4.5	953
C3	L4_scaffold20_RGP_0	47.42	Phage_integrase	T4SS_MOBP1	CMGI-3 (338)	4.5	953
C3	L3_scaffold23_RGP_0	47.42	Phage_integrase	T4SS_MOBP1	CMGI-3 (338)	4.5	953
C3	L20_scaffold19_RGP_0	47.42	Phage_integrase	T4SS_MOBP1	CMGI-3 (338)	4.5	953
C3	3614_scaffold19_RGP_0	47.42	Phage_integrase	T4SS_MOBP1	CMGI-3 (338)	4.5	953
C3	3492_scaffold30_RGP_0	47.42	Phage_integrase	T4SS_MOBP1	CMGI-3 (338)	4.5	953
C3	2975_scaffold16_RGP_0	47.42	Phage_integrase	T4SS_MOBP1	CMGI-3 (338)	4.5	953
C3	2971_scaffold15_RGP_0	47.42	Phage_integrase	T4SS_MOBP1	CMGI-3 (338)	4.5	953
C3	2969_scaffold13_RGP_0	47.42	Phage_integrase	T4SS_MOBP1	CMGI-3 (338)	4.5	953
C3	2968_scaffold16_RGP_0	47.42	Phage_integrase	T4SS_MOBP1	CMGI-3 (338)	4.5	953
C3	2951_scaffold17_RGP_0	47.42	Phage_integrase	T4SS_MOBP1	CMGI-3 (338)	4.5	953
C3	2945_scaffold15_RGP_0	47.42	Phage_integrase	T4SS_MOBP1	CMGI-3 (338)	4.5	953
C3	2930_scaffold18_RGP_0	47.42	Phage_integrase	T4SS_MOBP1	CMGI-3 (338)	4.5	953
C3	2895_scaffold12_RGP_0	47.42	Phage_integrase	T4SS_MOBP1	CMGI-3 (338)	4.5	953
C4	3610_scaffold19_RGP_0	40.31	Phage_integrase	T4SS_MOBP1	CMGI-3 (338)	4.5	953
C4	3454_scaffold14_RGP_0	40.31	Phage_integrase	T4SS_MOBP1	CMGI-3 (338)	4.5	953
C5	3614_scaffold30_RGP_1	31.02	Phage_integrase	T4SS_MOBP1	ICEVchBan9(16)	9.5	4884
C5	2975_scaffold28_RGP_1	31.02	Phage_integrase	T4SS_MOBP1	ICEVchBan9(16)	9.5	4884
C5	2971_scaffold27_RGP_2	31.02	Phage_integrase	T4SS_MOBP1	ICEVchBan9(16)	9.5	4884
C5	2969_scaffold26_RGP_1	31.02	Phage_integrase	T4SS_MOBP1	ICEVchBan9(16)	9.5	4884
C5	2968_scaffold28_RGP_0	31.02	Phage_integrase	T4SS_MOBP1	ICEVchBan9(16)	9.5	4884
C5	2951_scaffold20_RGP_0	31.02	Phage_integrase	T4SS_MOBP1	ICEVchBan9(16)	9.5	4884
C5	2945_scaffold20_RGP_0	31.02	Phage_integrase	T4SS_MOBP1	ICEVchBan9(16)	9.5	4884
C5	2895_scaffold25_RGP_1	31.02	Phage_integrase	T4SS_MOBP1	ICEVchBan9(16)	9.5	4884
C6	PP2-978_scaffold23_RGP_0	38.16	Phage_integrase	T4SS_MOBP1	ICEVchBan9(16)	10	5784
C6	PP2-843_contig_2_RGP_1	38.16	Phage_integrase	T4SS_MOBP1	ICEVchBan9(16)	10	5784
C6	2967_scaffold26_RGP_0	38.16	Phage_integrase	T4SS_MOBP1	ICEVchBan9(16)	10	5784
C7	07_121_1T1_scaffold13_RGP_2	7.86	Phage_integrase	T4SS_MOBV	-	-	-
C7	07_116_T1_scaffold11_RGP_2	7.86	Phage_integrase	T4SS_MOBV	-	-	-
C7	07_112_T1_scaffold17_RGP_2	7.86	Phage_integrase	T4SS_MOBV	-	-	-

C8	07_117_T1_scaffold15_RGP_0	15.40	Phage_integrase	profile_MOBM	ICEKpnSMU18037509-1(1388)	2.5	366
C9	07_038_2T2_scaffold28_RGP_0	15.10	Phage_integrase	profile_MOBM	ICETn4371_6067 (196)	2.5	449
C10	07_115_T2_scaffold37_RGP_0	13.94	Phage_integrase	profile_MOBM	IEVchRus1(590_IME)	2.5	437
C11	NPI-1_contig_1_pilon_RGP_5	13.60	Phage_integrase	profile_MOBM	IEVchRus1(590_IME	2.5	415
C12	07_117_T1_scaffold20_RGP_0	57.45	Phage_integrase	profile_MOBF	ICEVpaCan1(1071)	5.5	791
C13	07_115_T2_scaffold20_RGP_2	10.01	Phage_integrase	profile_MOBF	-	-	-
C14	07_116_T1_scaffold22_RGP_6	8.78	Phage_integrase	T4SS_MOBP1	-	-	-

Table S3. List of prophages identified from *V. europaeus* genomes, including clusters (A-J) and similar prophages.

**Cluster**	**Host**	**Phage code**	**Lenght (kb)**	**Match**	**Accession**	**Q cover**	**E- value**	**% Ident**
**A**	L2	VE-P42	41	Vibrio phage L9-1	OR762784.1	27%	0.0	85.40%
**B**	071316F	VE-P36	33.6	-	-	-	-	-
**C**	CECT8427	VE-P40	38.9	-	-	-	-	-
**C**	07_038_2T2	VE-P1	39.9	-	-	-	-	-
**C**	071316F	VE-P35	36.3	-	-	-	-	-
**D**	2967	VE-P16	46	-	-	-	-	-
**D**	PP2-978	VE-P49	46	-	-	-	-	-
**D**	2909	VE-P10	46	-	-	-	-	-
**D**	PP2-843	VE-P46	46	-	-	-	-	-
**D**	2974	VE-P23	46	-	-	-	-	-
**E**	CECT8136T	VE-P37	31.3	Vibrio phage 1.159.O._10N.261.46.F12	MG592527.1	77%	0.0	90.30%
**E**	PP-635	VE-P51	31.3	Vibrio phage 1.159.O._10N.261.46.F12	MG592527.1	77%	0.0	90.30%
**E**	PP-660	VE-P55	31.3	Vibrio phage 1.159.O._10N.261.46.F12	MG592527.1	77%	0.0	90.30%
**E**	PP-654	VE-P53	31.3	Vibrio phage 1.159.O._10N.261.46.F12	MG592527.1	77%	0.0	90.30%
**E**	PP2-843	VE-P47	31.9	Vibrio phage 1.159.O._10N.261.46.F12	MG592527.1	73%	0.0	95.51%
**E**	2967	VE-P15	31.9	Vibrio phage 1.159.O._10N.261.46.F12	MG592527.1	73%	0.0	95.51%
**E**	2974	VE-P24	31.9	Vibrio phage 1.159.O._10N.261.46.F12	MG592527.1	73%	0.0	95.51%
**E**	2909	VE-P9	31.9	Vibrio phage 1.159.O._10N.261.46.F12	MG592527.1	73%	0.0	95.51%
**E**	PP2-978	VE-P48	31.9	Vibrio phage 1.159.O._10N.261.46.F12	MG592527.1	73%	0.0	95.51%
**F**	L2	VE-P41	43.7	Vibrio phage ST2-2pr	MZ496294.1	22%	0.0	95.08%
**G**	3454	VE-P27	41.2	Vibrio phage ST2-2pr	MZ496294.1	25%	0.0	93.21%
**g**	2968	VE-P17	41.2	Vibrio phage ST2-2pr	MZ496294.1	25%	0.0	93.21%
**G**	3492	VE-P29	41.2	Vibrio phage ST2-2pr	MZ496294.1	25%	0.0	93.21%
**G**	3614	VE-P33	41.2	Vibrio phage ST2-2pr	MZ496294.1	25%	0.0	93.21%
**G**	2969	VE-P19	41.2	Vibrio phage ST2-2pr	MZ496294.1	25%	0.0	93.21%
**G**	2971	VE-P21	41.2	Vibrio phage ST2-2pr	MZ496294.1	25%	0.0	93.21%
**G**	2975	VE-P25	41.2	Vibrio phage ST2-2pr	MZ496294.1	25%	0.0	93.21%

**G**	3610	VE-P31	41.2	Vibrio phage ST2-2pr	MZ496294.1	25%	0.0	93.21%
**G**	2951	VE-P13	41.2	Vibrio phage ST2-2pr	MZ496294.1	25%	0.0	93.21%
H	07_116_T1	VE-P4	5.9	Inoviridae sp.	MH649021.1	6%	9,00E-17	71.91%
**I**	07_120_T1	VE-P6	8.5	-	-	-	-	-
**I**	CECT8426	VE-P39	8.5	-	-	-	-	-
**J**	L3	VE-P43	14.6	Inoviridae sp.	MH649021.1	0%	0.035	96.97%
**J**	L4	VE-P44	14.6	Inoviridae sp.	MH649021.1	0%	0.035	96.97%
**J**	L20	VE-P45	14.6	Inoviridae sp.	MH649021.1	0%	0.035	96.97%
**J**	07_121_1T1	VE-P7	6	Inoviridae sp.	MH649021.1	6%	9,00E-17	71.91%
**J**	07_110_T1	VE-P2	6	Inoviridae sp.	MH649021.1	6%	9,00E-17	71.91%
**J**	07_112_T1	VE-P3	6	Inoviridae sp.	MH649021.1	6%	9,00E-17	71.91%
**J**	07_116_T1	VE-P5	6	Inoviridae sp.	MH649021.1	6%	9,00E-17	71.91%
**J**	CECT8136T	VE-P38	8.4	Inoviridae sp.	MH649021.1	0%	0.007	96.97%
**J**	3614	VE-P34	8.4	Inoviridae sp.	MH649021.1	0%	0.007	96.97%
**J**	2895	VE-P8	8.4	Inoviridae sp.	MH649021.1	0%	0.007	96.97%
**J**	2945	VE-P12	8.4	Inoviridae sp.	MH649021.1	0%	0.007	96.97%
**J**	2969	VE-P20	8.4	Inoviridae sp.	MH649021.1	0%	0.007	96.97%
**J**	PP-660	VE-P54	8.4	Inoviridae sp.	MH649021.1	0%	0.007	96.97%
**J**	3492	VE-P30	8.4	Inoviridae sp.	MH649021.1	0%	0.007	96.97%
**J**	PP-654	VE-P52	8.4	Inoviridae sp.	MH649021.1	0%	0.007	96.97%
**J**	PP-635	VE-P50	8.4	Inoviridae sp.	MH649021.1	0%	0.007	96.97%
**J**	3454	VE-P28	8.4	Inoviridae sp.	MH649021.1	0%	0.007	96.97%
**J**	3610	VE-P32	8.4	Inoviridae sp.	MH649021.1	0%	0.007	96.97%
**J**	2930	VE-P11	8.4	Inoviridae sp.	MH649021.1	0%	0.007	96.97%
**J**	2975	VE-P26	8.4	Inoviridae sp.	MH649021.1	0%	0.007	96.97%
**J**	2968	VE-P18	8.4	Inoviridae sp.	MH649021.1	0%	0.007	96.97%
**J**	2951	VE-P14	8.4	Inoviridae sp.	MH649021.1	0%	0.007	96.97%
**J**	2971	VE-P22	8.4	Inoviridae sp.	MH649021.1	0%	0.007	96.97%

Table S4. RGP families identified from phage clusters.
Some prophages were associated with decontextualized RGPs, as is the case of prophages B (1/1), F (1/1), G (9/9), H (1/1), I (2/2) and J (6/23).

**Phage clusters**	**RGP families**
A	78.1
B	NS.55
C	21.4, 21.5, and 21.6
D	37.1
E	38, 51, and 53.1
F	NS.26
G	NS.2
H	RGP 3.1
J	RGP 3.1 and NS.9
I	NS.18

Table S5. Clonal strains identified within the *V. europaeus* collection from Rodriguez (2025). Core-genome SNPs differences among strains ranged from 0 to 28.

**Isolated from:**	**Location(s):**	**Host(s):**	**Cluster:**	**Clonal strains:**
Same bivalve species	Hatchery B (Spain)	*D. trunculus* (larvae and seawater)	Ia	3454, 3492
	Hatchery B (Spain)	*R. decussatus* (larvae and seawater)	Ia	2895, 2930, 2945, 2951, 2969, 2971, 2975
	Hatchery B (Spain)	*E. arcuatus* (larvae)	Ia	L3, L4
	Hatchery B (Spain)	*O. edulis* (larvae)	Ia	PP-654, PP-660
	Hatchery B (Spain)	*R. decussatus* (larvae)	Ib	2909, 2974, 2967
	Hatchery E (Spain)	*R. philippinarum* (spat)	Ib	PP2-843, PP2-978
	Hatchery D (France)	*M. gigas* (spat)	IIa	07/110 T1, 07/112 T1, 07/121 T1
	Hatchery D (France)	*M. gigas* (spat)	IIb	07/120 T1, CECT8426
Vertical transmission	Hatchery B (Spain)	*R. decussatus* broodstock to eggs	Ia	3610, 3614
Different hatcheries/hosts/dates	Hatchery E & B (Spain)	*R. philippinarum* vs. *R. decussatus*	Ib	PP2-978 vs. 2909/2974/2967
	Hatchery B (Spain)	*R. philippinarum* vs. *E. arcuatus*	Ia	L20 vs. L3/L4
	Hatchery B (Spain)	*D. trunculus* vs. *R. decussatus*	Ia	3454/3492 vs. 3610/3614
	Hatchery B (Spain)	*R. decussatus* vs. *D. trunculus*	Ia	2895/2930/2945/2951/2969/2971/2975 vs. 3454/3492
	Hatchery B (Spain)	*R. decussatus* (larvae) vs. *R. decussatus* (broodstock and eggs)	Ia	2895/2930/2945/2951/2969/2971/2975 vs. 3610/3614
	Hatchery B (Spain)	*O. edulis* vs. *R. philippinarum* vs. *E. arcuatus*	Ia	PP-654/PP-660 vs. L20; PP-654/PP-660 vs. L3/L4

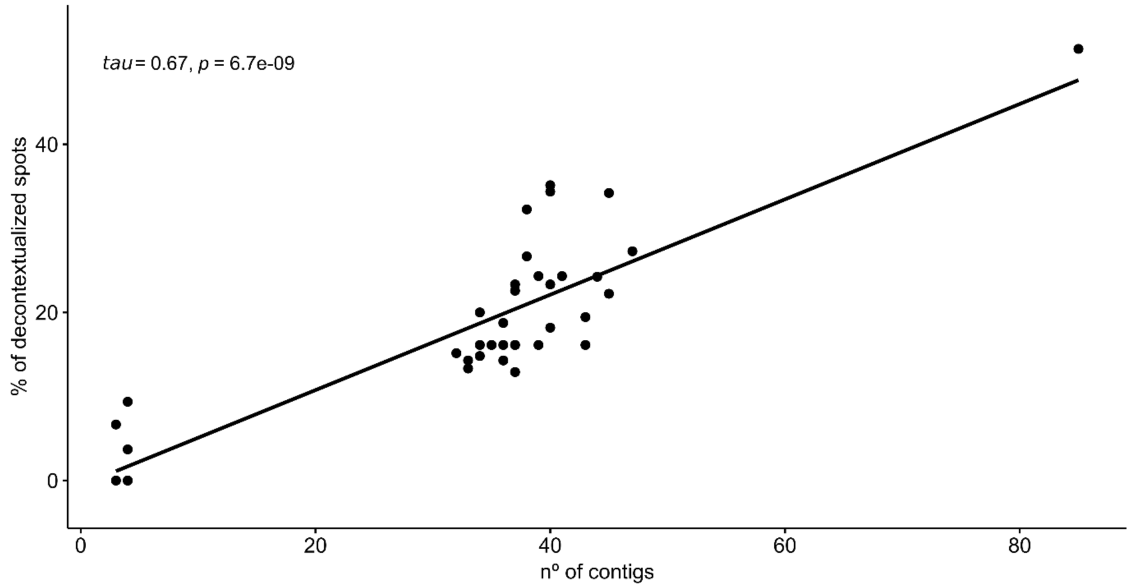

Supplementary Fig. S1. Correlation between the genome fragmentation and the number of decontextualized chromosomic RGPs. tau = 0.6738, p-value = 6.708e-09.
